# FluG and FluG-like FlrA Coregulate Manifold Gene Sets Vital for Fungal Insect-Pathogenic Lifestyle but Not Involved in Asexual Development

**DOI:** 10.1128/msystems.00318-22

**Published:** 2022-07-11

**Authors:** Chong-Tao Guo, Xin-Cheng Luo, Sen-Miao Tong, Yan Zhou, Sheng-Hua Ying, Ming-Guang Feng

**Affiliations:** a MOE Laboratory of Biosystems Homeostasis & Protection, Institute of Microbiology, College of Life Sciences, Zhejiang University, Hangzhou, Zhejiang, China; b College of Advanced Agricultural Sciences, Zhejiang A&F University, Hangzhou, Zhejiang, China; University of California San Diego

**Keywords:** entomopathogenic fungi, upstream developmental activation, genomic regulation, asexual development, spore quality, virulence, stress tolerance, upstream developmental regulator

## Abstract

The central developmental pathway (CDP) activator gene *brlA* is activated by the upstream genes *fluG* and *flbA*–*flbE* in Aspergillus nidulans. Increasing evidences of fungal genome divergence make it necessary to clarify whether such genetic principles fit Pezizomycotina. Previously, *fluG* disruption resulted in limited conidiation defect and little effect on the expression of *brlA* and *flbA*–*flbE* in Beauveria bassiana possessing the other FluG-like regulator FlrA. Here, single-disruption (SD) mutants of *flrA* and double-disruption (DD) mutants of *flrA* and *fluG* were analyzed to clarify whether FlrA and FluG are upstream regulators of key CDP genes. Despite similar subcellular localization, no protein-protein interaction was detected between FlrA and FluG, suggesting mutual independence. Three *flrA* SD mutants showed phenotypes similar to those previously described for Δ*fluG*, including limited conidiation defect, facilitated blastospore production, impaired spore quality, blocked host infection, delayed proliferation *in vivo*, attenuated virulence, and increased sensitivities to multiple stresses. Three DD mutants resembled the SD mutants in all phenotypes except more compromised pathogenicity and tolerance to heat shock- or calcofluor white-induced stress. No CDP gene appeared in 1,622 and 2,234 genes dysregulated in the Δ*flrA* and Δ*fluG* mutants, respectively. The majority (up/down ratio: 540:875) of those dysregulated genes were co-upregulated or co-downregulated at similar levels in the two mutants. These findings unravel novel roles for *flrA* and *fluG* in coregulating manifold gene sets vital for fungal adaptation to insect-pathogenic lifestyle and environment but not involved in CDP activation.

**IMPORTANCE** FluG is a core regulator upstream of central developmental pathway (CDP) in Aspergillus nidulans but multiple FluG-like regulators (FLRs) remain functionally uncharacterized in ascomycetes. Our previous study revealed no role for FluG in the CDP activation and an existence of sole FLR (FlrA) in an insect-pathogenic fungus. This study reveals a similarity of FlrA to FluG in domain architecture and subcellular localization. Experimental data from analyses of targeted single- and double-gene knockout mutants demonstrate similar roles of FrlA and FluG in stress tolerance and infection cycle but no role of either in CDP activation. Transcriptomic analyses reveal that FlrA and FluG coregulate a large number of same genes at similar levels. However, the regulated genes include no key CDP gene. These findings uncover that FlrA and FluG play similar roles in the fungal adaptation to insect-pathogenic lifestyle and environment but no role in the activation of CDP.

## INTRODUCTION

Understanding asexual development-activating mechanism in fungal insect pathogens is of special importance for designing and improving production technology of high-quality conidia as active ingredients of fungal pesticides (1–3). In Aspergillus nidulans, central developmental pathway (CDP) regulate aerial conidiation and is activated by upstream developmental activation pathway (UDAP), in which *fluG* and *flbA* to *flbE* function (4–6). The formation of phialides and chained conidia relies upon sequential activation of the CDP genes *brlA*, *abaA*, and *wetA* (7–10). The UDAP genes were found in early studies on repressive fluffy mutations, which repressed *brlA* function and conidiation (11–14). Such “fluffy” genes have been characterized as players in the activation of *brlA* expression by three *fluG*-cored cascades, namely, *fluG-flbA*, *fluG-flbC*, and *fluG-flbE/flbB/flbD* (15–23). As a core UDAP regulator, *fluG* is required for initiation of conidiation (11). Its role stems from its mediating the synthesis of an endogenous diffusible factor, which acts as an essential signal to initiate asexual development upon accumulation onto aerial hyphae (24). The repressors of conidiation include SfgA, NsdD and G-proteins (25–27). More recent analysis of FluG sequence has revealed an essentiality of its C-terminal γ-glutamyl ligase region for asexual development and an interesting link between this region and N-terminal amidohydrolase region (28). Indeed, many more non-UDAP genes are actively involved in fungal conidiation, including light-responsive regulators (29–31). Even in aspergilli, *fluG* does not necessarily play the same regulatory role as in A. nidulans. For instance, conidiation was reduced, but not abolished, by deletion of *fluG* in Aspergillus flavus (32), and not affected by deletion of *fluG* in Aspergillus niger (33). Due to increasing evidences of genome divergence in ascomycetes, it is necessary to clarify whether the genetic control principles on asexual development of A. nidulans fit Pezizomycotina (6, 31, 34, 35). The necessity is strengthened by the existence of FluG homologs with molecular sizes of 860 to 914 (large-molecule type) and 437 to 534 (small-molecule type) amino acids (aa) and of multiple FluG-like regulators (FRLs) (36). It remains intangible whether and how *fluG* and its analogs function in UDAP to activate the *brlA* expression for commencement of asexual development in different lineages of ascomycetes.

In Beauveria bassiana as a main source of wide-spectrum fungal pesticides safe to honeybees (1, 37), the key CDP genes *brlA* and *abaA* act as master regulators of asexual developmental processes, including aerial conidiation, submerged blastospore production, and dimorphic (hypha-blastospore) transition required for yeast-like proliferation in insect hemocoel to accelerate mycosis development and host death because such processes were all abolished in the absence of *brlA* or *abaA* (38). The other CDP gene *wetA* and the downstream *vosA* also have proved essential for B. bassiana’s conidiation and conidial maturation (39). In our recent study, all CDP genes and *flbA* to *flbE* in B. bassiana were active in the absence of *fluG*, resulting in only a 10% decrease in conidial yield (36). A sharp increase of blastospore production in the submerged Δ*fluG* cultures correlated well with upregulated expression of both *brlA* and *abaA* presumably associated with earlier upregulation of most *flb* genes and of the other FluG-like regulator (FLR) gene, suggesting a likelihood that this FLR might act as an alternative player in the fungal UDAP. Singular deletion mutants of *flbA*–*flbE* also showed limited or little conidiation defect (40). Our genome survey revealed the existence of one to four FLRs annotated as putative glutamine synthetases or hypothetical proteins in different fungi. However, such FLRs have never been investigated in ascomycetes, making it unclear whether FLRs function like FluG in fungal UDAP. This study seeks to characterize sole FLR (named FlrA) in B. bassiana using single-disruption (SD) mutants of *flrA* and double-disruption (DD) mutants of both *flrA* and *fluG*. An emphasis is placed upon the impacts of SD and DD on the time course expression levels of all *flb* and CDP genes in plate and submerged cultures and the production of aerial conidia and submerged blastospores. Our goal is to clarify whether FlrA and FluG are independent or collaborative players in UDAP, whether FlrA and FluG activate the expression of key CDP genes, and whether the genetic control principles of asexual development elucidated in A. nidulans are applicable to B. bassiana. As presented below, like *fluG*, *flrA* was essential in the fungal insect-pathogenic lifestyle but not involved in CDP activation.

## RESULTS

### Sequence comparison and phylogenetic links of fungal FLR and FluG homologs.

**FIG 1:**
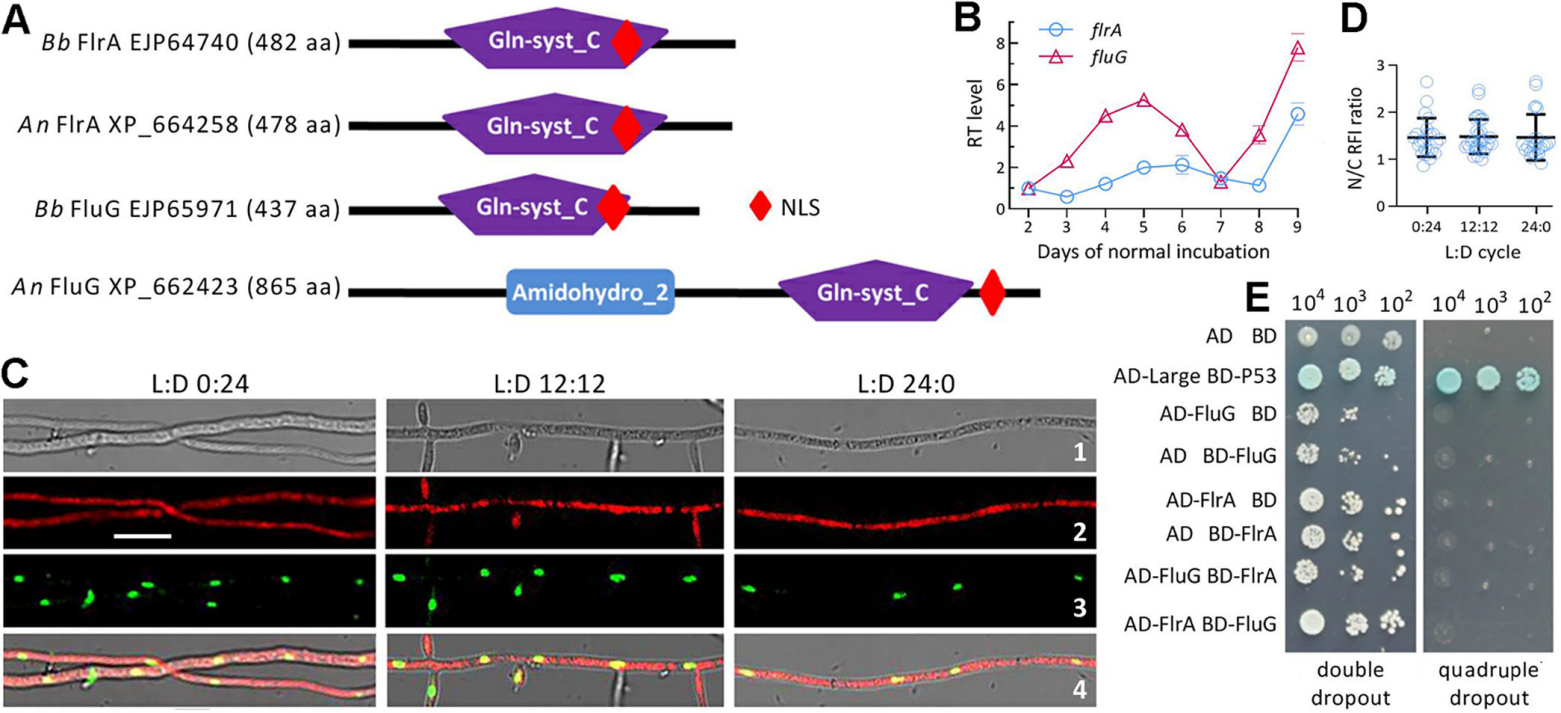
Transcription profile and subcellular localization of FlrA and its relationship with FluG in B. bassiana. (A) Sequence comparison of B. bassiana (*Bb*) FlrA and FluG with A. nidulans (*An*) counterparts. Domains and NLS motif were predicted from each protein at http://smart.embl-heidelberg.de/ and https://nls-mapper.iab.keio.ac.jp/cgi-bin/NLS_Mapper_form.cgi, respectively. (B) Relative transcript (RT) levels of *flrA* and *fluG* in the SDAY cultures of wild-type (WT) *Bb* strain during a 7-day incubation at the optimal regime of 25°C and L:D 12:12 with respect to the standard level on day 2. (C) LSCM images (scale bars: 5 μm) for subcellular localization of red fluorescence-tagged FlrA fusion protein expressed in the WT strain. Cell samples were taken from the 3-day-old SDBY cultures grown at 25°C in the L:D cycles of 0:24, 12:12, and 24:0 and stained with the nuclear dye DAPI (shown in green). Bright, expressed, stained and merged views of the same field are shown in images 1, 2, 3, and 4, respectively. (D) Nuclear versus cytoplasmic red fluorescence intensity (N/C-RFI) ratios of the fusion protein in the hyphal cells. Error bars denote standard deviations (SDs) of the means from three cDNA samples analyzed via qPCR (B) or 23 to 32 cells in the examined hyphae (D). (E) Y2H assay for an interaction of FlrA with FluG (AD-FlrA BD-FluG) or of FluG with FlrA (AD-FluG BD-FlrA). Note that the constructed diploid cells except positive control (AD-Large BD-P53) were unable to grow on the quadruple-dropout plate.

The BLASTp search with the query FlrA sequence of B. bassiana resulted in recognition of both FLR and FluG homologs in selected ascomycetous fungi including aspergilli. One to four more FLRs were found in most examined fungi (Table S1). Typical FLRs were clustered to a clade distinctive from that of FluG homologs (Fig. S1). Exceptionally, the query shows higher sequence identity (74% to 98%) and closer phylogenetic link to small-molecule FluG homologs in some FRL-deficient fungi than to large-molecule FluG homologs coexisting with FLRs in many other fungi. In domain architecture, FlrA homologs feature sole glutamine synthetase-catalytic domain (Gln-syst_C) as does FluG in B. bassiana or sole FLR in A. nidulans, whose large-molecule FluG shares N-terminal Amidohydro_2 and C-terminal Gln-syst_C domains with homologs in other fungi (Fig. 1A; Table S1). Associated with the catalytic domain is a nuclear localization signal (NLS) motif predicted from each protein sequence.

10.1128/msystems.00318-22.1 FIG S1 Phylogenetic analysis of FluG (in red) and FlrA (in black) homologues in 20 filamentous fungi including insect and noninsect pathogens. The tree is constructed using the maximum likelihood method in MEGA7 at http://www.megasoftware.net/. Bootstrap values of 1,000 replications are shown at nodes. Scale: branch length proportional to genetic distance. The NCBI accession code of each protein, the length of its amino acid sequence, and its protein sequence identity to the homologue of B. bassiana FlrA are given in the parentheses following the fungal name. Download FIG S1, JPG file, 1.5 MB. Copyright © 2022 Guo et al. 2022 Guo et al. https://creativecommons.org/licenses/by/4.0/ This content is distributed under the terms of the Creative Commons Attribution 4.0 International license.

10.1128/msystems.00318-22.4 TABLE S1 Comparison of domains predicted from FluG and FluG-like regulator (FLR) homologs of some insect and noninsect pathogenic ascomycetes (*Shown in red and black are FluG and FLR homologs, respectively). Download Table S1, JPG file, 1.4 MB. Copyright © 2022 Guo et al. 2022 Guo et al. https://creativecommons.org/licenses/by/4.0/ This content is distributed under the terms of the Creative Commons Attribution 4.0 International license.

### Transcription profile, subcellular localization, and functional independence of FlrA.

Transcript levels of *flrA* and *fluG* in the wild-type (WT) strain B. bassiana ARSEF 2860 (designated WT) showed similar fluctuating trends during a 9-day incubation on SDAY (Sabouraud dextrose agar plus yeast extract) plates at the optimal regime of 25°C and L:D (light/dark) 12:12 (Fig. 1B). The fusion protein FlrA-mCherry accumulated in hyphal cytoplasm and nuclei (Fig. 1C). Nuclear versus cytoplasmic red fluorescence intensity (N/C-RFI) ratios assessed from the hyphal cells were averagely 1.45 to 1.48 in three L:D cycles (*F*_2,75_ = 0.037, *P = *0.964; Fig. 1D). This nucleocytoplasmic shuttling status of FlrA is similar to that of FluG previously observed in B. bassiana (36).

Yeast two-hybrid (Y2H) assay revealed no evidence for an interaction between FlrA and FluG due to an inability for the diploids AD-FlrA-BD-FluG and AD-FluG-BD-FlrA to grow on the quadruple-dropout plate (Fig. 1E). This suggests their mutual independence in B. bassiana.

### FlrA is dispensable for radial growth but essential for stress tolerance.

**FIG 2:**
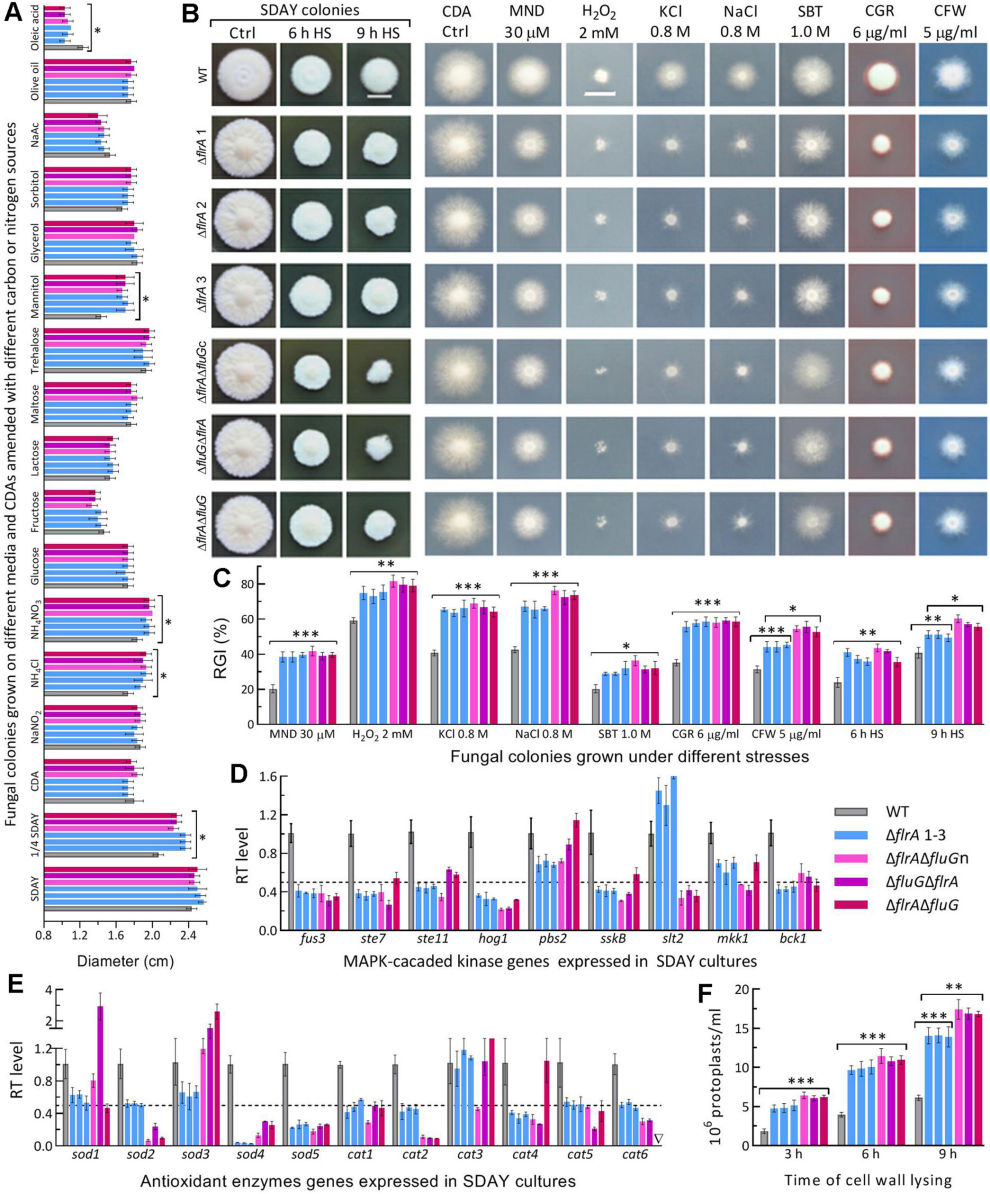
Radial growth rates of B. bassiana in the absence of *flrA* and of both *flrA* and *fluG*. (A) Diameters of fungal colonies grown at the optimal regime of 25°C and L:D 12:12 for 7 days on the plates of rich medium SDAY, 1/4 SDAY, minimal medium CDA, and CDAs amended with different carbon/nitrogen sources. (B, C) Images (scale bar: 10 mm) and relative growth inhibition (RGI) percentages of fungal colonies incubated at 25°C for 7 days on CDA plates supplemented with indicated concentrations of menadione (MND), H_2_O_2_, KCl, NaCl, sorbitol (SBT), Congo red (CGR) and calcofluor white (CFW), and of SDAY colonies incubated at 25°C for 5-day growth recovery after 2-day-old colonies were exposed to a 42°C heat shock (HS) for 6 h and 9 h, respectively. Each colony was initiated by spotting 1 μL of a 10^6^ conidia/mL suspension. (D, E) Relative transcript (RT) levels of MAPK-cascaded and antioxidant enzyme genes in the 3-day-old SDAY cultures of mutants with respect to the WT standard. The dashed line denotes a significant level of 1-fold downregulation. (F) Concentrations of protoplasts released from cell suspensions after 6 h and 9 h of cell wall lysing with enzymes in 1 M NaCl at 37°C. *P < *0.05*; 0.01**; or 0.001*** in Tukey’s HSD tests. Error bars: SDs of the means from three independent replicates.

Three SD mutants and three DD mutants created in different strategies (Fig. S2; Table S2) grew as well as WT on different media (SDAY, 1/4 SDAY, CDA [Czapek-Dox agar]) at the optimal regime (Fig. S3). No fluffy phenotype was observed in their colonies. Instead, their SDAY colonies showed similar folds as seen in the previous SD mutants of the MAPK/Slt2-cascaded kinase genes required for cell-wall integrity (41), and a pigmentation pattern similar to, but lighter than, that of WT. All SD and DD mutants were significantly facilitated in radial growth on 1/4 SDAY and CDA amended with NH_4_Cl or NH_4_NO_3_ as sole nitrogen source and with mannitol as sole carbon source (Fig. 2A). However, they showed little growth defects on SDAY and CDAs containing other nitrogen or carbon sources except oleic acid.

10.1128/msystems.00318-22.2 FIG S2 Generation and identification of *flrA* and *fluG* mutants in B. bassiana. (A to C) Schematic diagram for the disruption strategy of *flrA* and identification of its mutants via PCR and qPCR analyses, respectively. The PCR-detected DNA fragments are 1,896 bp for three Δ*fluG* mutants and 1,442 bp for the wild-type (WT) strain, resulting in disruption of *flrA* by deleting a partial promoter/coding fragment of 472 bp (1,442 + 926 **–** 1,896 = 472) from WT as illustrated in the diagram and its transcript hardly detected in the 3-day-old SDAY and TPB cultures of each Δ*flrA* mutant. (D to F) Schematic diagrams for three double disruption (DD) strategies of *flrA* and *fluG*. (G) Identification of DD mutants via PCR analysis. The PCR-detected bands indicate successful deletion of partial promoter/coding fragment of *fluG* from Δ*flrA* (1,368 + 1,436 **–** 2,427 = 377 bp for Δ*flrA*Δ*fluG*n), full-length coding and partial flanking regions of *flrA* from Δ*fluG* (2,904 + 1,436 **–** 1,880 = 2,460 bp for Δ*fluG*Δ*flrA*) or full-length coding and partial flanking regions of *fluG* from Δ*flrA* (3,085 + 1,436 **–** 1,983 = 2,528 bp for Δ*flrA*Δ*fluG*). (H, I) Relative transcript (RT) levels of *flrA* and *fluG* in the 3-day-old SDAY and TPB cultures of three DD mutants with respect to the WT standard. ML, molecular ladder of genomic DNA (B, G). Error bars (C, H, and I), standard deviations of the means from three cDNA samples derived from independent cultures of each strain. Download FIG S2, JPG file, 0.7 MB. Copyright © 2022 Guo et al. 2022 Guo et al. https://creativecommons.org/licenses/by/4.0/ This content is distributed under the terms of the Creative Commons Attribution 4.0 International license.

10.1128/msystems.00318-22.3 FIG S3 Upside and backside views of fungal colonies grown on SDAY, 1/4 SDAY, and CDA plates for 7 days at the optimal regime of 25°C and L:D 12:12 after initiated by spotting 1 μL aliquots of a 10^6^ conidia/mL suspension. Download FIG S3, JPG file, 1.4 MB. Copyright © 2022 Guo et al. 2022 Guo et al. https://creativecommons.org/licenses/by/4.0/ This content is distributed under the terms of the Creative Commons Attribution 4.0 International license.

10.1128/msystems.00318-22.5 TABLE S2 Paired primers used for manipulation of *flrA* and *fluG* in B. bassiana (*The underlined regions denote the restriction enzyme sites for the fusion of *flrA* cDNA (*Xma*I/*Bam*HI) to *mCherry*, the deletion of partial *flrA* (*Xma*I/*Sac*I and *Xba*I/*Hpa*I) from the WT strain and the deletion of full-length *fluG or flrA* (*Xba*I/*Eco*RI and *Spe*I/*Eco*RV) from Δ*flrA* or Δ*fluG*. The underlined and bold regions denote the restriction enzyme sites for ligating *fluG* or *flrA* cDNA (*Eco*RI/*Bam*HI) to pGADT7 and pGBKT7 for Y2H assay, respectively). Download Table S2, JPG file, 1.6 MB. Copyright © 2022 Guo et al. 2022 Guo et al. https://creativecommons.org/licenses/by/4.0/ This content is distributed under the terms of the Creative Commons Attribution 4.0 International license.

The SD and DD mutants’ growths were markedly suppressed by oxidants, osmotic agents and cell wall perturbing agents added to CDA; their growths on SDAY were also suppressed by 6- or 9-h heat shock (Fig. 2B). Compared with WT, the mutants were significantly (10% to 32%) more sensitive to all tested stresses (Fig. 2C). The DD mutants were significantly more sensitive to calcofluor white or 9-h heat shock than the SD mutants.

Most kinase genes in MAPK cascades required for regulation of multiple stress responses (41–43) were suppressed significantly in the SD and DD mutants (Fig. 2D). Their increased sensitivities to two oxidants correlated with repressed expression of key antioxidant enzyme genes (Fig. 2E), including *sod2* as a major contributor to total SOD activity (44) and *cat2/catB* and *cat5/catP* crucial to total catalase activity (45). These genes were more downregulated in the DD mutants. The mutants’ cell wall damages were further presented by easier lysis of their cells, which released 1.3- to 2.3-fold more protoplasts than the WT cells treated with cell wall lysing enzymes (Fig. 2F). The DD mutants’ cells released significantly more protoplasts than did the SD mutants’ cells after 9-h treatment.

The SD and DD mutants’ phenotypes were similar, though not identical, to those observed previously in the Δ*fluG* mutant (36). These data indicate that *flrA* and *fluG* play similar roles of in B. bassiana’s responses to various stresses but dispensable role in normal growth.

### Greater role of *flrA* in conidial quality control than in aerial conidiation.

**FIG 3:**
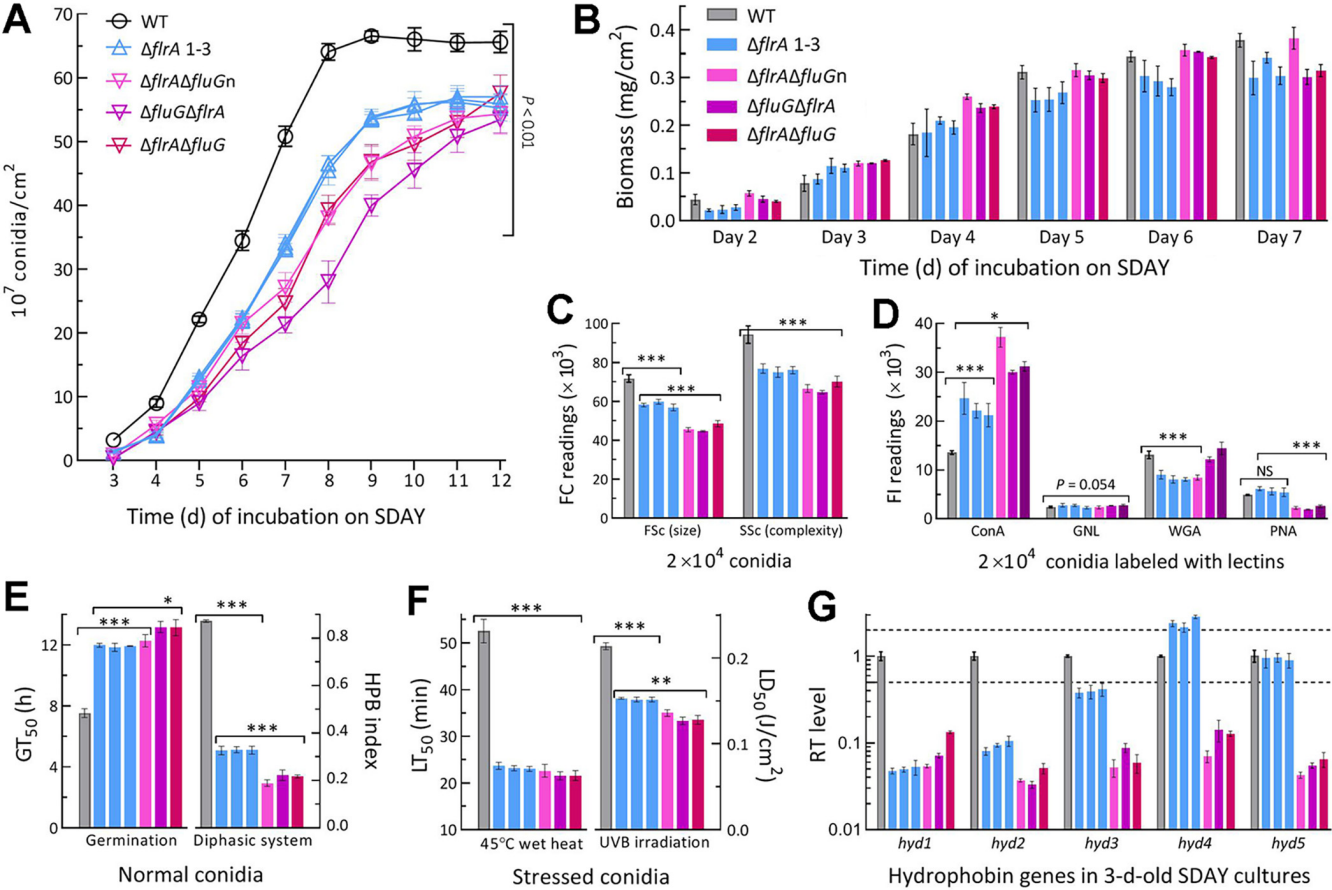
Aerial conidiation and conidial quality of B. bassiana in the absence of *flrA* and of both *flrA* and *fluG*. (A, B) Conidial yields and biomass levels measured from the SDAY cultures during a 12-day incubation at the optimal regime of 25°C and L:D 12:12, respectively. The cultures were initiated by spreading 100-μL aliquots of a 10^7^ conidia/mL suspension. (C) Mean size and complexity (density) of conidia denoted by the FSc and SSc readings in the flow cytometry (FC) of 2 × 10^4^ conidia (per sample). (D) Fluorescence intensity (FL) readings indicating the contents of hydrocarbon epitopes on the surfaces of 2 × 10^4^ conidia (per sample) labeled with the fluorescent lectins ConA, WGA, GNL and PNA, respectively. (E) Conidial GT_50_ (h) assessed at 25°C and hydrophobicity (HPB) index assessed in a diphasic (aqueous-organic) system. (F) LT_50_ (min) for conidial tolerance to a 45°C wet-heat stress and LD_50_ (J/cm^2^) for conidial resistance to UVB irradiation. (G) Relative transcript (RT) levels of five hydrophobin family genes in the 3-day-old SDAY cultures of all mutants with respect to the WT standard. Upper and lower dashed lines denote significant levels of 1-fold up- and downregulation, respectively. *P < *0.05*; 0.01**; or 0.001*** in Tukey’s HSD tests. Error bars: SDs of the means from three independent replicates.

The WT cultures initiated by spreading 100-μL conidial suspension aliquots at the optimal regime usually starts conidiation on day 3 and reach a maximum of conidial yield within 8 days (46, 47). Conidial yields of all SD and DD mutants were measurable in 3-day-old SDAY cultures and decreased significantly by 57% (±7.3) and 83% (±11.3), respectively, compared with the WT yield (Fig. 3A). The SD and DD mutants’ yield reductions diminished to 42% (±2.7) and 55% (±7.0) on day 5, 28% (±2.5%) and 45% (±10.2) on day 8, 16% (±2.3) and 26% (±5.5) on day 10, and 15% (±2.8) and 16% (±4.4) on day 12, respectively. Biomass levels assessed from cellophane-overlaid SDAY cultures (Fig. 3B) revealed no link of the early more suppressed conidiation to biomass accumulation in the mutants’ cultures.

Moreover, the SD and DD mutants were compromised severely in conidial quality. In flow cytometry, conidial size and complexity (density) denoted by the readings of forward scatter (FSc) and side scatter (SSc) detectors were reduced by 18.8% (±2.6) and 18.5% (±2.3) for the SD mutants and 36% (±6.0) and 29% (±3.8) for the DD mutants relative to WT (Fig. 3C). Fluorescence-activated cell sorter (FACS) analysis of lectin-labeled conidia revealed differential changes of hydrocarbon epitopes on the SD or DD mutants’ conidial surfaces, including increased contents of α-glucose and α-*N*-acetylglucosamine (GlcNAc) labeled by concanavalin A (ConA) and reduced contents of β-GlcNAc and sialic acid residues labeled by wheat germ agglutinin (WGA) and of β-galactose residues labeled by peanut agglutinin (PNA) (Fig. 3D). The DD mutants were significantly more impaired than the SD mutants in conidial viability (GT_50_ at 25°) and hydrophobicity (Fig. 3E). Conidial heat tolerance and UVB resistance were also lowered by 56% and 29% for the SD mutants and 58% and 39% for the DD mutants, respectively (Fig. 3F). The SD and DD mutants’ difference in hydrophobicity reduction correlated well with transcriptional repression of five (89% to 96%) and three (61% to 96%) hydrophobin-coding genes, respectively (Fig. 3G), including *hyd1* and *hyd2* required for conidial hydrophobicity and adherence to insect cuticle (48).

**FIG 4:**
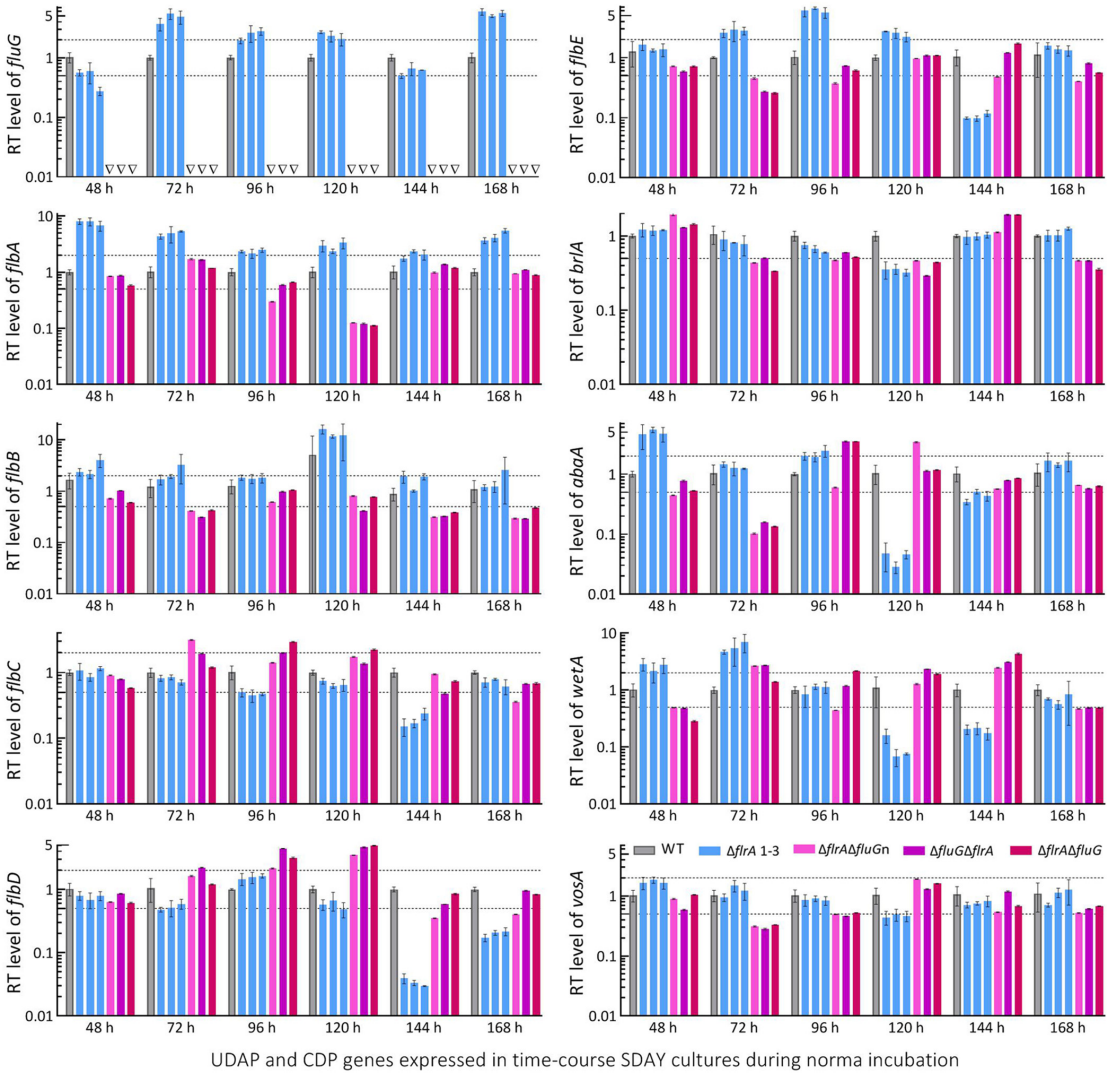
Relative transcript (RT) levels of putative UDAP (*fluG* and *flbA* to *flbE*) and confirmed CDP (*brlA*, *abaA*, and *wetA* plus *vosA*) genes affected by disruption of *flrA* and double disruption of *flrA* and *fluG* in B. bassiana. The cDNA samples were derived daily from the SDAY cultures of each strain during a 7-day incubation at the optimal regime of 25°C and L:D 12:12 and subjected to qPCR analysis. The upper and lower dashed lines denote significant levels of 1-fold up- and downregulation with respect to the WT standard, respectively. Error bars: SDs of the means from three independent cDNA samples per strain.

Further, real-time quantitative PCR (qPCR) analysis with paired primers (Table S3) was conducted to reveal time course expression levels of *fluG*, *flbA–flbE*, and three CDP genes and downstream *vosA* required for the fungal conidiation and conidial maturation (38, 39). Differential expression levels of *fluG* and five *flb* genes were observed in the SD cultures during a 7-day incubation at the optimal regime (Fig. 4). In the DD mutants, expression of *fluG* was abolished while each *flb* gene remained differentially expressed in a time course manner. The CDP genes significantly repressed in the SD cultures included *brlA* and *vosA* on day 4, and *abaA* and *wetA* on days 4 and 5, but their expressions were markedly upregulated or unaffected at the remaining time points. Likewise, the CDP genes were differentially expressed in the DD mutants’ cultures during the incubation period.

10.1128/msystems.00318-22.6 TABLE S3 Paired primers used for qPCR analysis of phenotype-related genes in B. bassiana. Download Table S3, JPG file, 1.7 MB. Copyright © 2022 Guo et al. 2022 Guo et al. https://creativecommons.org/licenses/by/4.0/ This content is distributed under the terms of the Creative Commons Attribution 4.0 International license.

Altogether, all *flb* and CDP genes remained active in the SD and DD mutants as did the previous Δ*fluG* mutant (36), excluding a role of FlrA or FluG in activating key CDP genes to initiate conidiation. The previous and present studies unraveled much greater importance of FlrA or FluG for conidial quality control than conidiation in B. bassiana.

### Blastospore production facilitated in the absence of *flrA* or both *flrA* and *fluG*.

**FIG 5:**
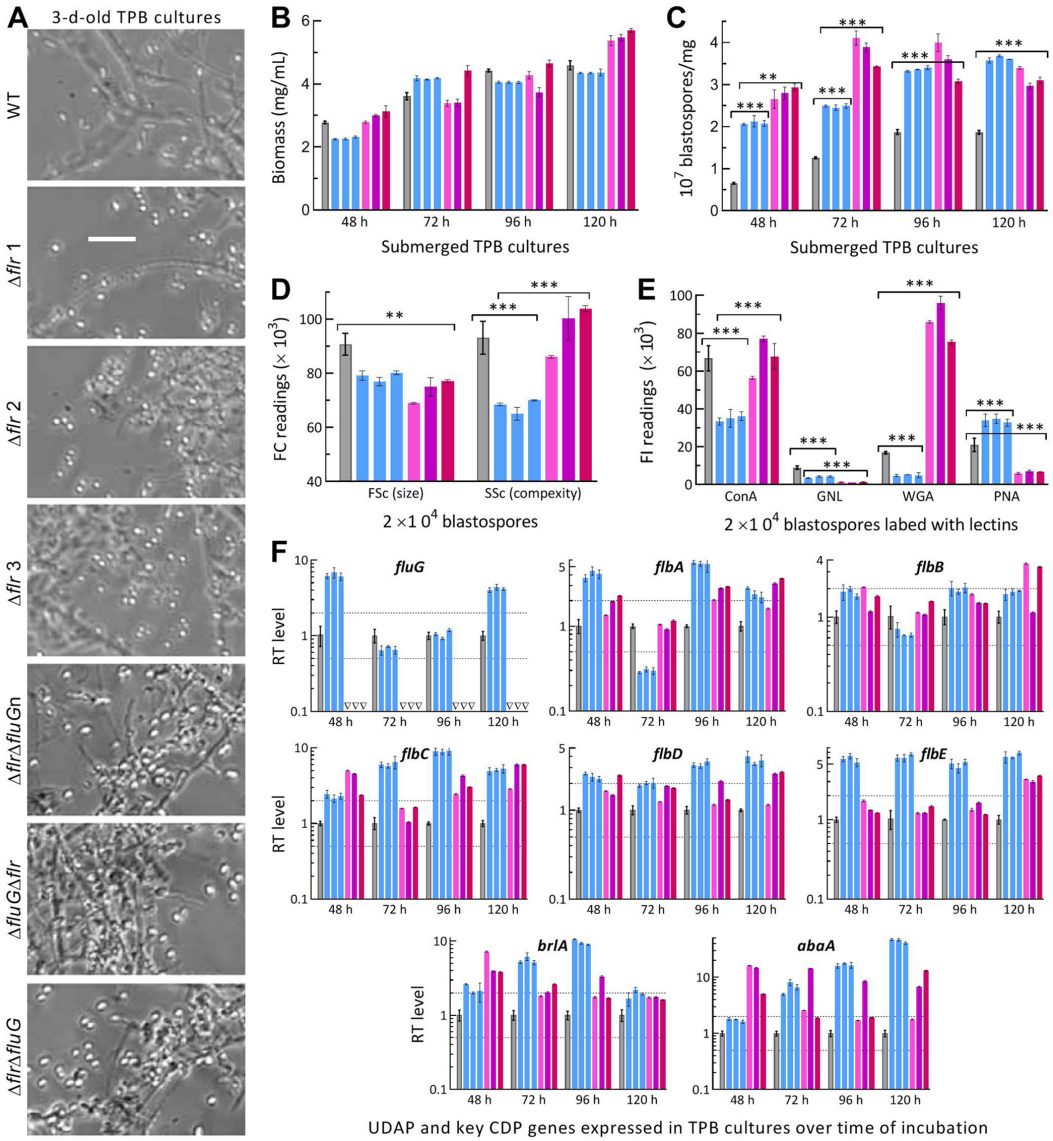
Submerged blastospore production affected by disruption of *flrA* and double disruption of *flrA* and *fluG* in B. bassiana. (A) Microscopic images (scale bar: 20 μm) for a status of blastospore production in the 3-day-old cultures of a 10^6^ conidia/mL TPB mimicking insect hemolymph. (B, C) Biomass levels and dimorphic transition rates measured from the TPB cultures during a 5-day incubation at 25°C, respectively. (D) Mean size and complexity of blastospores indicated by the FSc and SSc readings in flow cytometry (FC) of 2 × 10^4^ blastospores (per sample) from the 3-day-old TPB cultures. (E) Fluorescence intensity (FL) readings indicating the contents of hydrocarbon epitopes on the surfaces of 2 × 10^4^ blastospores (per sample) labeled with the fluorescent lectins ConA, WGA, GNL, and PNA, respectively. (F) Relative transcript (RT) levels of putative UDAP (*fluG* and *flbA* to *flbE*) and key CDP (*brlA* and *abaA*) genes in the TPB cultures of all mutants with respect to the WT standard during the 5-day incubation. Upper and lower dashed lines denote significant levels of 1-fold up- and downregulation, respectively. *P < *0.05*, 0.01**, or 0.001*** in Tukey’s HSD tests. Error bars: SDs of the means from three independent replicates.

Like conidiation, submerged blastospore production mimicking proliferation *in vivo* is controlled by *brlA* or *abaA* in B. bassiana (38). Previously, it was described that both *brlA* and *abaA* were greatly upregulated in the Δ*fluG* cultures grown in trehalose-peptone broth (TPB) mimicking insect hemolymph, leading to a drastic increase in blastospore production through dimorphic transition (36). A similar situation was observed in the SD and DD mutants’ TPB cultures (Fig. 5A). Their biomass levels were similar or close to the WT levels during a 5-day incubation (Fig. 5B). Compared with WT, the SD and DD mutants showed blastospore yields sharply enhanced by 219% and 328% at 48 h, 97% and 202% at 72 h, 80% and 90% at 96 h, and 94% and 69% at 120 h, respectively (Fig. 5C). Moreover, their blastospore size diminished significantly despite differential changes in complexity (Fig. 5D). Hydrocarbon epitopes on the surfaces of their blastospores labeled by ConA, WGA, PNA, and Galanthus nivalis lectin (GNL, specific to mannose residues) also were differentially altered (Fig. 5E). Transcriptional analysis revealed similar time course changes of *fluG* and *flbA–flbE* in all mutants’ TPB cultures (Fig. 5F). However, both *brlA* and *abaA* were upregulated consistently in their TPB cultures at all sampling time points.

These data highlighted great facilitation of blastospore production and highly active status of *brlA* and *abaA* in the SD and DD mutants’ TPB cultures as seen previously in the TPB cultures of Δ*fluG* (36). The results excluded again a role of either FrlA or FluG in the activation of key CDP genes to mediate blastospore production in B. bassiana, suggesting that some other pathways mediate the fungal *flb* and CDP genes in the absence of *flrA* alone or both *flrA* and *fluG*.

### Essential role of *flrA* in fungal insect pathogenicity and hemocoel colonization.

**FIG 6:**
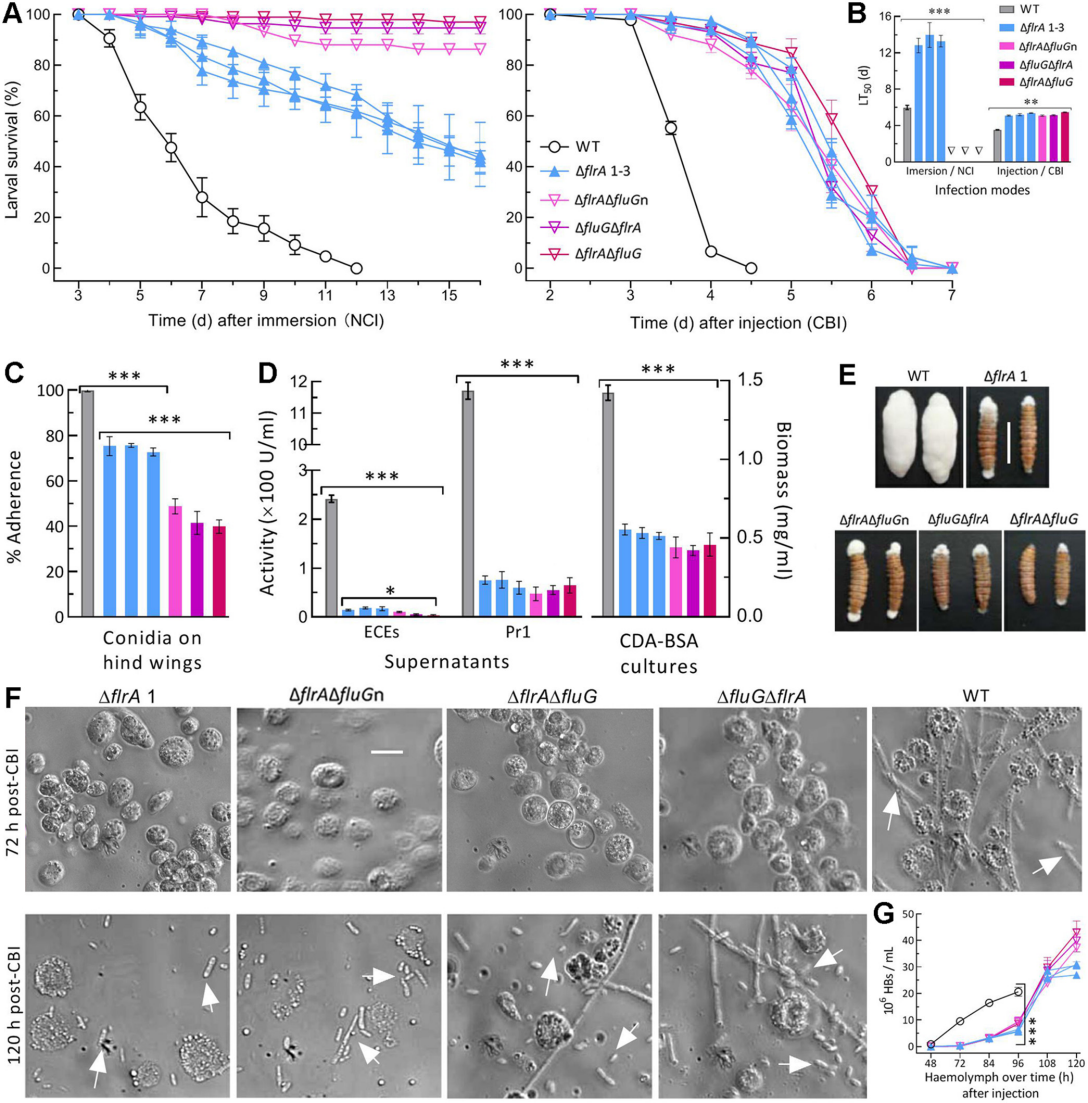
Indispensability of *flrA* and *fluG* for insect pathogenicity and virulence-related cellular events of B. bassiana. (A, B) Time-survival trends of G. mellonella larvae after topical application (immersion) of a 10^7^ conidia/mL suspension for normal cuticle infection (NCI) and intrahemocoel injection of ~500 conidia per larva for cuticle-bypassing infection (CBI) and LT_50_ estimates (d) from the trends. (C) Conidial adherence to locust hind wing cuticle assessed as percent ratios of postwash counts over prewash counts with respect to the WT standard. (D) Biomass levels and total ECEs and Pr1 activities (U/mL) assessed from 3-day-old CDB-BSA cultures and their supernatants, respectively. (E) Images (scale bar: 10 mm) for fungal outgrowths on the surfaces of insect cadavers 6 days after death from CBI. (F, G) Microscopic images (scale bar: 20 μm) for the status of hyphal bodies (HBs, arrowed) and host hemocytes (spherical or subspherical cells) in the hemolymph samples taken from surviving larvae 72-h and 120-h post-CBI and concentrations of hyphal bodies in the samples taken 48–120 h post-CBI, respectively. *P < *0.05*, 0.01**, or 0.001*** in Tukey’s HSD tests. Error bars: SDs of the means from three independent replicates.

The WT strain caused 100% mortality of Galleria mellonella larvae within 12 days via normal cuticle infection (NCI) or 5 days via cuticle-bypassing infection (CBI/injection) (Fig. 6A). The SD and DD mutants caused mean mortalities of no more than 60% and 10% 16 day post-NCI although their CBI killed all tested larvae within 7 days. Consequently, NCI resulted in mean (±SD) LT_50_ of 6.0 (±0.24) days for WT and of 13.4 (±0.99) days for the SD mutants but no computable LT_50_ for the DD mutants against the model insect (Fig. 6B). The SD and DD mutants’ LT_50_s via CBI were prolonged by ~48% (~1.7 days) relative to the WT’s 3.5 days.

Next, cellular events crucial for fungal NCI and subsequent hemocoel colonization were examined. First, conidial adherence to locust wing cuticle decreased significantly by 26% (±2.7) and 57% (±10.4) for the SD and DD mutants compared to WT (Fig. 6C). For all tested strains, the estimates of conidial adherence were linearly correlated with hydrophobicity indices (r^2^ = 0.71, *F*_1,19_ = 46.4, *P < *0.0001). Second, total activities of extracellular enzymes (ECEs) and Pr1 proteases required for successful NCI (49, 50) were reduced by 93% (±1.1) and 97% (±2.4) in the supernatants from the 3-day-old CDB-BSA cultures of the SD mutants and 94% (±1.3) and 95% (±1.2) in the DD mutants’ supernatants, respectively (Fig. 6D, left panel). The two reductions diminished to 81% and 84% for the SD mutants and to 92% and 85% for the DD mutants by deducting the effect of decreased biomass accumulation (Fig. 6D, right panel). Third, an impaired capability of the mutants’ penetrating insect cuticle was shown by fungal outgrowths on cadaver surfaces. The WT strain formed a heavy layer of hyphal outgrowth completely covering the cadavers 6-day postdeath (Fig. 6E). However, none of the mutants was able to grow directly out of the cadaver surfaces by cuticle penetration from host hemocoel. Instead, their sparse outgrowths were strictly restrained to mouthparts and anuses, leading to a “bold” phenotype on most cadaver surfaces. Finally, a status of hemocoel localization by yeast-like budding to speed up host mummification was revealed by microscopic examination of hemolymph samples taken from surviving larvae. Hyphal bodies were abundant for WT at 72-h postinjection but were hardly observed for all mutants (Fig. 6F). Consequently, mean concentration of WT-formed hyphal bodies in the samples was 0.83 × 10^6^ cells/mL at 48 h, rapidly increased to 9.5 × 10^6^ cells/mL at 72 h, and reached 20.6 × 10^6^ cells/mL at 96 h, followed by hemolymph depletion (Fig. 6G). In contrast, the SD and DD mutants’ counts were unavailable at 48 h, 0.36 × 10^6^ and 0.11 × 10^6^ cells/mL at 72 h, and sharply increased to 29.5 × 10^6^ and 40.1 × 0^6^ cells/mL at 120 h, respectively.

These data indicated an essentiality of *flrA* for B. bassiana’s NCI and hemocoel colonization and reinforced the same role of *fluG* as elucidated previously (36). More reduced insect pathogenicity of the DD mutants than of the SD mutants via NCI was attributable to their differential defects in conidial hydrophobicity and adherence. For all SD and DD mutants, similarly attenuated virulence via CBI was mainly due to at least 1-day delayed formation and proliferation of hyphal bodies in insect hemocoel.

### Transcriptomic insight into similar functions of *flrA* and *fluG*.

The Δ*flrA*- and Δ*fluG*-specific transcriptomes contained 1,622 and 2,234 differentially expressed genes (DEGs. up/down ratios: 635:987 and 780:1454; the same meaning for all ratios mentioned below), respectively. Intriguingly, 1,415 genes (540:875) taking 13.65% in the fungal genome (51) were individually co-up- or co-downregulated at similar levels in the two mutants (Table S4), highlighting overlapping roles of *flrA* and *fluG* in genomic regulation.

10.1128/msystems.00318-22.7 TABLE S4 A list of 1,415 genes codysregulated in the Δ*flrA* and Δ*fluG* mutants of B. bassiana. Download Table S4, XLSX file, 0.1 MB. Copyright © 2022 Guo et al. 2022 Guo et al. https://creativecommons.org/licenses/by/4.0/ This content is distributed under the terms of the Creative Commons Attribution 4.0 International license.

**FIG 7:**
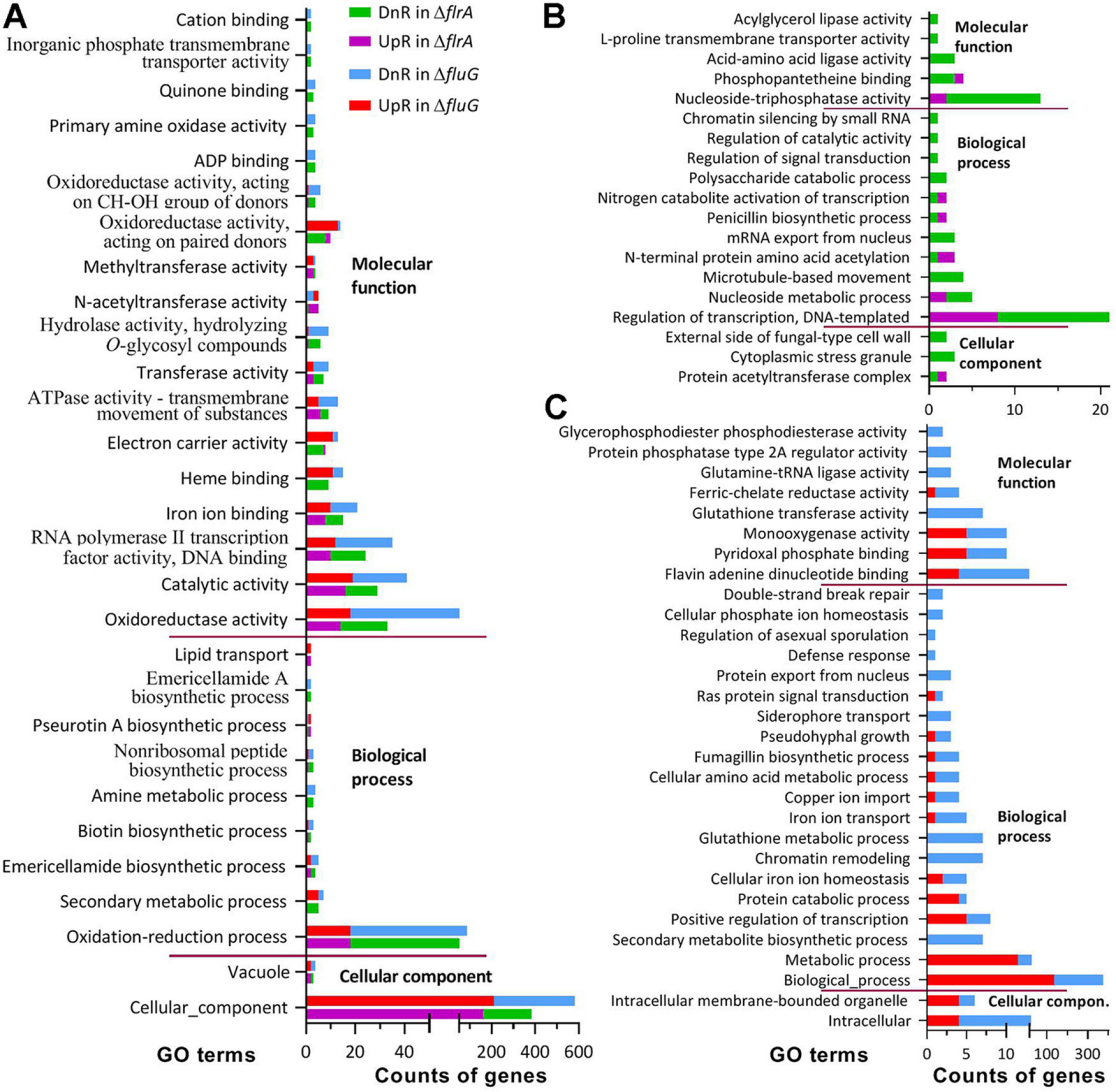
Counts of significantly upregulated (UpR) and downregulated (DnR) genes enriched to GO terms of three function classes in the Δ*frlA*- and Δ*fluG*-specific transcriptomes of B. bassiana at the significant level of *P < *0.05. (A) Counts of dysregulated genes enriched to the same GO terms of Δ*frlA* and Δ*fluG*. (B, C) Counts of genes enriched to the Δ*frlA*- and Δ*fluG*-specific GO terms, respectively.

Gene ontology (GO) analysis resulted in 1,018 (322:696) and 2,052 DEGs (548:1504) enriched to 48 and 59 GO terms of three function classes in Δ*flrA* and Δ*fluG,* respectively (Tables S5). The two mutants shared 29 terms, including two terms of cellular component, eight terms of biological process, and 18 terms of molecular function (Fig. 7A). These terms comprised 922 (292:628) and 1,206 DEGs (358:948) in Δ*flrA* and Δ*fluG,* respectively. The remaining terms individually contained no more than 10 DEGs despite fewer exceptions in Δ*flrA* (Fig. 7B) than in Δ*fluG* (Fig. 7C). Almost all enriched GO terms featured low up/down ratios in each mutant and were functionally repressed. The terms co-repressed in Δ*flrA* and Δ*fluG* included cellular component (163:383 and 210:581), oxidation-reduction process (18:52 and 18:86), oxidoreducase activity (13:33 and 18:53), catalytic activity (16:29 and 19:41), RNA polymerase II transcription factor activity (10:24 and 12:35), iron ion binding (8:15 and 10:21), ATPase activity (6:9 and 5:13), transferase activity (3:7 and 3:9), and hydrolase activity (1:6 and 1:9).

10.1128/msystems.00318-22.8 TABLE S5 Summation of GO function classes and terms enriched to the Δ*flrA* and Δ*fluG* mutants of B. bassiana. Download Table S5, XLSX file, 0.01 MB. Copyright © 2022 Guo et al. 2022 Guo et al. https://creativecommons.org/licenses/by/4.0/ This content is distributed under the terms of the Creative Commons Attribution 4.0 International license.

**FIG 8:**
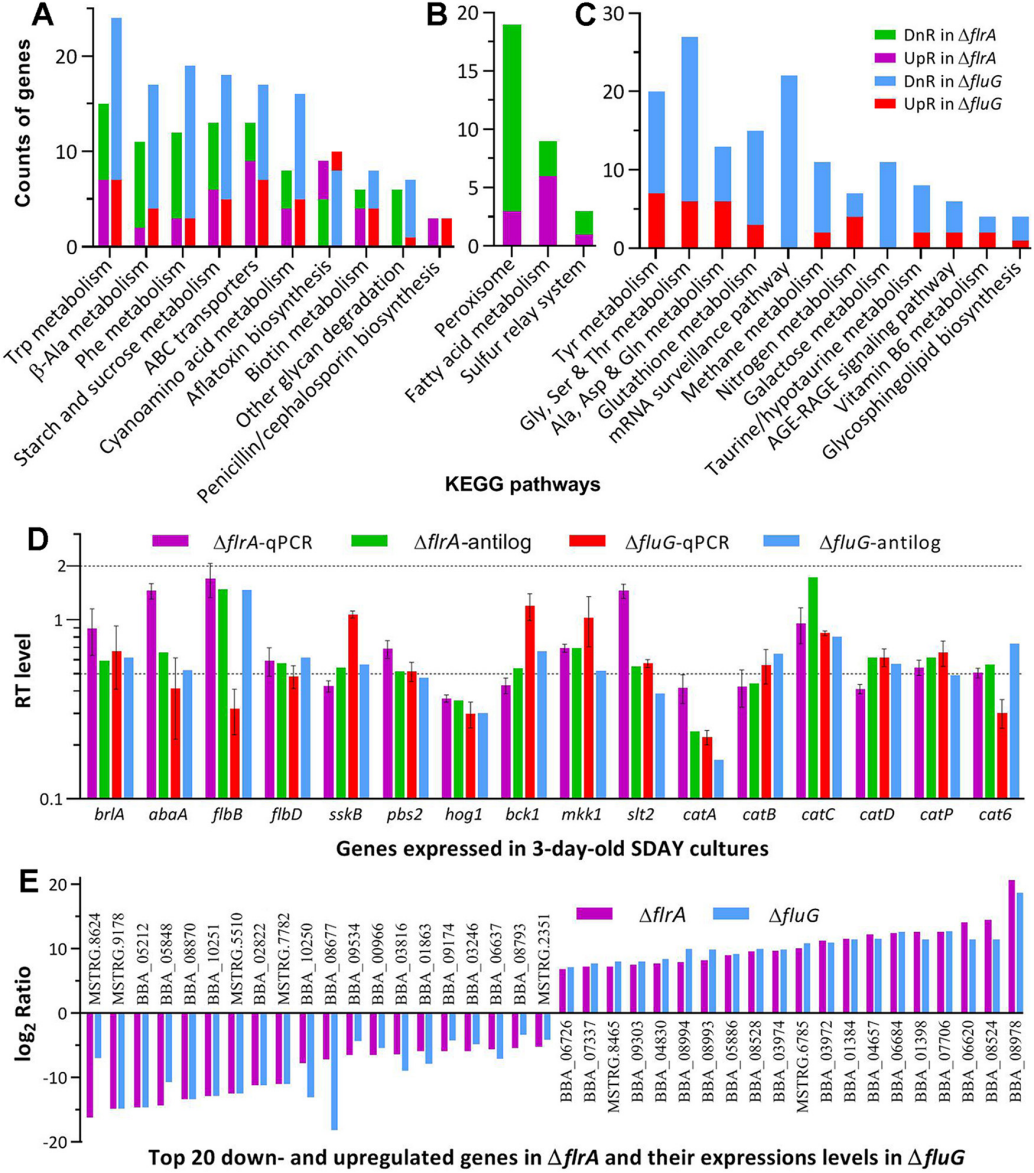
Pathway and validity analyses of B. bassiana Δ*frlA*- and Δ*fluG*-specific transcriptomes. (A to C) Counts of significantly upregulated (UpR) and downregulated (DnR) genes enriched to the same KEGG pathways of both Δ*frlA* and Δ*fluG* mutants and different pathways of each mutant at the significant levels of *P < *0.05, respectively. (D) Relative transcript (RT) levels of 16 qPCR-analyzed genes in the 3-day-old SDAY cultures of Δ*frlA* and Δ*fluG* versus WT with their anti-log_2_ ratio values in the transcriptomes of the two mutants. Error bars: SDs of the means from three independent cDNA samples analyzed via qPCR. (E) The log_2_ ratio values of top 20 down- and upregulated genes in the Δ*frlA* and Δ*fluG* mutants (see Table S4 for all co-up- or co-downregulated genes in the null mutants).

In the Kyoto Encyclopedia of Genes and Genomes (KEGG) analysis, the Δ*flrA* and Δ*fluG* mutants had 177 (57:120) and 386 (84:282) DEGs enriched to 13 and 22 pathways, respectively (Table S6). Of those, 10 pathways were shared by Δ*flrA* (47:89) and Δ*fluG* (49:134) (Fig. 8A). Most of the shared pathways were virtually repressed, including carbon/nitrogen metabolisms, ABC transporters, biotin metabolism, and other glycan degradation, because each was dominated by downregulated genes in the two mutants. Exceptionally, two shared pathways responsible for biosyntheses of aflatoxin and penicillin/cephalosporin were upregulated. Three pathways were specifically repressed in Δ*flrA* (Fig. 8B), including peroxisome (3:19), fatty acid metabolism (6:9), and sulfur relay system (1:3). The Δ*fluG* mutant had more pathways specifically repressed (Fig. 8C), including metabolisms of various amino acids (19:60), glutathione metabolism (3:15), mRNA surveillance (0:22), methane metabolism (2:11), nitrogen metabolism (4:7), galactose metabolism (0:11), taurine/hypotaurine metabolism (2:8), and glycosphingolipid biosynthesis (1:4).

10.1128/msystems.00318-22.9 TABLE S6 Summation of KEGG pathways enriched to the Δ*flrA* and Δ*fluG* mutants of B. bassiana. Download Table S6, XLSX file, 0.01 MB. Copyright © 2022 Guo et al. 2022 Guo et al. https://creativecommons.org/licenses/by/4.0/ This content is distributed under the terms of the Creative Commons Attribution 4.0 International license.

The transcriptomes were validated by comparing anti-log_2_ *R* values with relative transcript levels of 16 qPCR-analyzed genes, which showed similar transcript trends (Fig. 8C). Moreover, most of top 20 co-up- or co-downregulated genes in Δ*flrA* and Δ*fluG* were overlapped (Fig. 8D), reinforcing transcriptomic validity and similar role for *flrA* and *fluG* in global gene mediation.

There were 883 co-dysregulated genes annotatable in function. Listed in Table S7 are annotatable genes associated with Δ*flrA* and Δ*fluG* phenotypes. First, none of the CDP genes required for B. bassiana conidiation (38, 39) was co-dysregulated. Instead, two other seemingly development-related but functionally uncharacterized genes were co-up- (BBA_02427) or co-downregulated (BBA_03814), giving no clue to an *flrA* or *fluG* role in CDP activation. Second, 24 co-dysregulated genes (9:15) were associated with the fungal NCI and virulence. Particularly, the coding genes of bassianolide nonribosomal peptide synthetase (BBA_02630) and beauvericin biosynthetic protein (BBA_09727) reported as virulence factors (52, 53) were greatly corepressed. Third, the two mutants’ increased sensitivities to oxidative, cell wall perturbing, and osmotic and thermal stresses correlated well with 32 codysregulated genes (8:24) involved in cell wall integrity, 22 (3:19) involved in response to oxidative stress, seven (2:5) involved in response to heat shock, 53 (21:32) involved in cellular transport, homeostasis and multidrug resistance, and 22 (5:17) involved in stress-responsive signal transduction. Fourth, many more codysregulated genes (170:417) were involved in carbon and nitrogen metabolisms, energy conversion, biogenesis, and secondary metabolism. Finally, many codysregulated genes were involved in direct/indirect transcription regulation because they encode transcription factors (20:40) and enzymes/proteins involved in posttranslational modifications (17:14) or in RNA/DNA processing and chromatin remodeling events (4:39). Notably, most of these transcription regulators were corepressed in the Δ*flrA* and Δ*fluG* mutants, providing an insight into the repression of most enriched GO terms and KEGG pathways.

10.1128/msystems.00318-22.10 TABLE S7 Codysregulated genes associated with phenotypes in the Δ*flrA* and Δ*fluG* mutants of B. bassiana. Download Table S7, XLSX file, 0.01 MB. Copyright © 2022 Guo et al. 2022 Guo et al. https://creativecommons.org/licenses/by/4.0/ This content is distributed under the terms of the Creative Commons Attribution 4.0 International license.

## DISCUSSION

In B. bassiana, conidiation and blastospore production are CDP-governed developmental processes required for NCI and hemococoel colonization (38, 47, 54).The SD and DD mutants exhibited no fluffy phenotype, limited conidiation defects, and facilitated blastospore production as did the previous Δ*fluG* (36), coinciding well with time course transcription profiles of their key CDP genes in plate and submerged cultures. The transcription profiles of all *flb* and CDP genes in the SD and DD mutants exclude a role for FluG or FrlA in the fungal UDAP. Therefore, the cascades controlling asexual development in fungal pathogens that have adapted to wide-spectrum or specific hosts and environments may not necessarily show the same structure as in A. nidulans (4–6). This could be true for small-molecule FluG homologs lacking an N-terminal Amidohydro_2 domain. Despite dispensable role in vegetative growth and CDP activation, FlrA and FluG are functionally similar and essential for B. bassiana’s fitness to insect-pathogenic life cycle and host habitats.

First, *flrA* and *fluG* are essential for B. bassiana’s NCI and insect pathogenicity. This is reinforced by the SD and DD mutants’ NCI severely compromised and nearly abolished, respectively, due to differential defects in conidial hydrophobicity and adherence to insect cuticle and drastically decreased secretion of cuticle-degrading enzymes (49, 50). Previously, *hyd1* and *hyd2* were shown to mediate biosyntheses of classes I and II hydrophobins and their assembly into conidial coat determinant to conidial hydrophobicity and adherence (48). Among three adhesin genes (*adh1* to *3*), only *adh2* proved functional in NCI due to *hyd1* expression repressed by its deletion (55). Drastic repression or abolished expression of *hyd1* and *hyd2* was often linked to reduced hydrophobicity/adherence and severely hindered or even abolished NCI among certain genes studied in B. bassiana (56–59). In this study, *hyd1* and *hyd2* were among three and five hydrophobin genes differentially repressed in the SD and DD mutants, respectively, providing an explanation for more compromised NCI of the DD mutants than of the SD mutants.

Moreover, successful NCI results in hyphal invasion into host hemocoel, where hyphae form hyphal bodies via CDP-governed dimorphic transition to accelerate host mummification (38). In the dying insect, hyphal bodies turn back into hyphae to penetrate the host cuticle for outgrowth and conidiation on cadaver surfaces (54). Such dimorphic transition was blocked in the SD and DD mutants. This is well demonstrated by their LT_50_s prolonged similarly via CBI due to more than 1-day delay of proliferation *in vivo* and an incapability of their penetrating host cuticle for outgrowth. Previously, injected conidia were evidently encapsulated by aggregated host hemocytes for first 48 h, and then appeared in the form of hyphal bodies (60). Breaking the encapsulation relies upon fungal ability to collapse host immune defense, which generates reactive oxygen species including superoxide radical anions and H_2_O_2_ (61). The delayed proliferation *in vivo* implicates that the SD and DD mutants could take longer reaction time to collapse the host immune system. This implication is verified by the mutants’ impaired conidia and defects in stress responses regulated by MAPK cascades (61). Intriguingly, repressed expression of their MAPK kinase genes concurred with differential repression of antioxidant enzyme genes crucial to decomposition of superoxide anions and H_2_O_2_ (44, 45). These suggest a role of FlrA in mediating expression of stress-responsive signaling and effector genes as did FluG previously (36). Notably, the SD and DD mutants’ conidia and blastospores were smaller in size and altered in hydrocarbon epitopes, which comprise pathogen-associated molecule patterns (PAMPs) to be perceived by host PAMP recognition receptors (62). The altered hydrocarbon epitopes implicate that the mutants’ conidia injected could be more readily perceived by the host receptors, inducing stronger encapsulation to be longer broken. Once the host immune defense was collapsed, the mutants’ cells proliferated *in vivo* as rapidly as seen in their TPB cultures, in which *brlA* and *abaA* were consistently upregulated. Therefore, FlrA and FluG play similar roles in collapsing host immune defense for hemocoel colonization by B. bassiana after NCI.

Our trancscriptomic analysis revealed largely overlapping roles for FlrA and FluG in comediating 1,415 genes. The majority of them were individually downregulated at similar levels in Δ*flrA* and Δ*fluG*, correlating with the mutants’ phenotypes. As examples, their increased sensitivities to various stresses were obviously due to the low up/down ratios of those genes involved in antioxidant activity, cell wall integrity, heat shock response, and cellular transport and homeostasis critical for multiple stress responses. The mutants’ virulence loss was associated with malfunction of those genes involved in cuticle-degrading hydrolase activity, antioxidant activity crucial for response to host immune defense (63), and syntheses of bassianolide and beauvericin as virulence factors (52, 53). Notably, the GO terms corepressed in Δ*flrA* and Δ*fluG* included the activities of RNA polymerase II transcription factor as a core mediator of signal transduction (64), and of multiple transferases involved in posttranslational modifications and chromatin remodeling, which are vital for transcriptional regulation (65, 66). The corepressed GO terms offer an answer to largely overlapped regulatory roles of FlrA and FluG in B. bassiana.

Finally, our qPCR and transcriptomic analyses revealed no role for either FluG or FlrA in mediating the expression of key CDP genes. In B. bassiana, *brlA* and *abaA* function as master regulators of asexual development (38) as seen in A. nidulans (7–10) and are speculated to be mediated by multiple routes. For instance, sharply repressed or nearly abolished expression of *brlA* and/or *abaA* is often associated with severe or extremely severe conidiation defects in the SD mutants of several genes involved in different pathways. The studied genes encode histone acetyltransferases and deacetylases (67–70), histone lysine methyltransferases (59, 71, 72), components of MAPK/Fus3 cascade (42, 43), blue-light receptor VVD (46), frequency proteins Frq1 and Frq2 (47, 52), vacuolar protein VLP4 (73), lysyl-tRNA synthetase KRS (74), cyclophilin B CypB (75), and carbon catabolite repressor Cre1 (76). Among those, Gcn5-acetylated histone H3K14 was proven to act as an epigenetic mark binding to the promoter of *brlA* for its activation (67). Opposite rhythms of Frq1 and Frq2 in nucleus enable persistent activation of CDP genes in a circadian day to orchestrate nonrhythmic conidiation that leads to rapid maximization of conidial yield regardless of photoperiod change (47, 49). In Metarhizium *robertsii*, the CDP activator AbaA mediates conidiation by its binding to the *veA* promoter (77). These studies suggest diverse routes of CDP activation in insect-pathogenic fungi other than the FluG-cored cascades documented in A. nidulans (4–6).

Conclusively, FluG and FlrA play similar roles in B. bassiana’s fitness to insect-pathogenic lifestyle and environment but nor role in the fungal UDAP. Our findings offer a novel insight into markedly overlapping roles for FlrA and FluG in regulating genomic expression and biological aspects.

## MATERIALS AND METHODS

### Bioinformatic analysis of fungal FLR and FluG homologs.

The amino acid sequence of B. bassiana FlrA (EJP64740) revealed in the previous study (36) was used as a query to search through the NCBI databases of some ascomycetous fungi including insect pathogens and noninsect pathogens via online BLASTp analysis (https://blast.ncbi.nlm.nih.gov/Blast.cgi). Phylogenetic linkages of the query with identified FlrA homologs were analyzed using a maximum likelihood method in online MEGA7 program (http://www.megasoftware.net/). Conserved domains predicted from the FlrA and FluG (EJP65971) sequences of B. bassiana at http://smart.embl-heidelberg.de/ were compared with those of their homologs in other fungi, followed by predicting an NLS motif from each protein sequence at https://nls-mapper.iab.keio.ac.jp/cgi-bin/NLS_Mapper_form.cgi.

### Subcellular localization of FlrA in B. bassiana.

Red fluorescence-tagged FlrA fusion protein was expressed in the WT strain as described previously for construction of transgenic strain expressing FluG-GFP fusion protein with the vector pAN52-C-gfp-bar (36), where C denotes the cassette 5′-*Pme*I-SpeI-EcoRV-EcoRI-BamHI-3′ driven by homologous promoter P*tef1*. Briefly, the vector was modified by replacing *gfp* with *mCherry* (KC294599). The coding sequence of *flrA* was amplified from the WT cDNA with paired primers (Table S2) and ligated to N-terminus of *mCherry* using a one-step cloning kit (Vazyme, Nanjin, China). The vector pAN52-flrA-mCherry-bar was integrated into the WT strain via *Agrobacterium* mediated transformation. Putative transformants were screened by the *bar* resistance to phosphinothricin (200 μg/mL). A transformant showing desired red fluorescence signal was grown on SDAY (4% glucose, 1% peptone, and 1.5% agar plus 1% yeast extract) for conidiation. The resultant conidia were suspended in SDBY (i.e., agar-free SDAY) and incubated at 25°C for 3 day in the L:D cycles of 0:24, 12:12, and 24:0 on a shaking bed (150 rpm). Hyphal samples from the cultures were stained with the nuclear dye DAPI (4′,6′-diamidine-2′-phenylindole dihydrochloride; Sigma-Aldrich, Shanghai, China) and visualized through laser scanning confocal microscopy (LSCM). The ImageJ software (https://imagej.nih.gov/ij/) was used to measure red fluorescence intensities from a fixed circular area moving in the cytoplasm and nucleus of each of 23 to 32 cells in the hyphae from the culture grown in each L:D cycle. The measurements were used to compute N/C-RFI ratios as relative accumulation levels of expressed FlrA-mCherry fusion protein in the nuclei of hyphal cells.

### Y2H assay for FlrA-FluG interaction.

To reveal whether FlrA works alone or together with FluG, Y2H assay was performed as described elsewhere (78). Briefly, the coding sequence of *flrA* or *fluG* amplified from the WT cDNA was inserted into the prey vector pGADT7 (AD) and the bait vector pGBKT7 (BD), respectively. After verification by sequencing, the constructs were transformed into the strains Saccharomyces cerevisiae Y187 and Y2HGold, respectively, followed by 24 h of pairwise yeast mating at 30°C on YPD (1% yeast extract, 2% peptone, 2% glucose plus 0.04% adenine hemisulfate salt). The diploids AD-FlrA-BD-FluG and AD-FluG-BD-FlrA were screened in parallel with positive control (AD-LargeT-BD-P53) and negative controls (AD-BD and the constructs AD-FlrA-BD, AD-BD-FlrA, AD-FluG-BD, and AD-BD-FluG) on the double-dropout (SDM/-Leu/-Trp/X-α- Gal/AbA) and quadruple-dropout (SDM/-Leu/-Trp/-Ade/-His/X-α-Gal/AbA) plates. All yeast colonies were initiated by spotting 10^4^, 10^3^, and 10^2^ cells, respectively, and incubated at 30°C for 3 days.

### Generation of *flrA* and *fluG* mutants.

The disruption strategy of *fluG* (BBA_04942) in the previous study (36) was used to generate disruption mutants of *flrA* (BBA_06309) by deleting an N-terminal partial promoter/coding DNA fragment of 472 bp from the WT genome through homologous recombination of its 5′ flanking and 3′ coding/flanking DNA fragments, which were separated by the *bar* marker in the vector p0380-5′flrA-bar-3′flrA (Fig. S2A). The vector was integrated into the WT strain as aforementioned. Putative mutant colonies were screened by the *bar* resistance to phosphinothricin (200 μg/mL), followed by verification of recombination events through PCR (Fig. S2B) and qPCR analyses. Paired primers used for the amplification of DNA fragments and the detection of targeted DNA and cDNA samples are listed in Table S2. Due to repeated failures to complement *flrA* into an identified Δ*flrA* mutant in many attempts, three SD mutants (Δ*flrA* 1 to 3) showing abolished *flrA* expression (Fig. S2C) were used in the study.

The DD mutants of *flrA* and *fluG* were created by deleting an N-terminal promoter/coding DNA fragment (377 bp; designated *fluG*n) of *fluG* from the identified mutant Δ*flrA* 1, a full-length coding and partial flanking fragment (2,460 bp) of *flrA* from the previous Δ*fluG* mutant, and a full-length coding and partial flanking fragment (2,538 bp) of *fluG* from the Δ*flrA* mutant (Fig. S2D to F), respectively. DD was achieved by homologous recombination of 5′ and 3′ DNA fragments of a target gene separated by *nat1* marker in the vector p0380-5′*x*-nat1-3′*x* (*x* = *fluG* or *flrA*), which was ectopically integrated into the SD mutant of the other target gene as aforementioned. Putative mutant colonies were screened by the *nat1* resistance to nourseothricin (50 μg/mL). Their recombinant events were verified through PCR (Fig. S2G) and qPCR analyses with paired primers (Table S2). The DD mutants Δ*flrA*Δ*fluG*n, Δ*fluG*Δ*flrA*, and Δ*flrA*Δ*fluG* with the expression of either target gene being abolished or hardly detectable in both plate and submerged cultures (Fig. S2H and I) were evaluated in parallel with the SD mutants and the parental WT in the following experiments of three independent replicates unless specified otherwise.

### Assays for radial growth rates under normal conditions and stresses.

Fungal colonies were initiated by spotting 1 μL aliquots of a 10^6^ conidia/mL suspension on the plates of SDAY, 1/4 SDAY, CDA (3% sucrose, 0.3% NaNO_3_, 0.1% K_2_HPO_4_, 0.05% KCl, 0.05% MgSO_4_, and 0.001% FeSO_4_ plus 1.5% agar) and CDAs amended with different carbon (glucose, trehalose, fructose, lactose, maltose, mannitol, glycerol, sodium acetate, olive oil, and oleic acid) or nitrogen (NaNO_2_, NH_4_Cl, and NH_4_NO_3_) sources. After a 7-day incubation at the optimal regime of 25°C and L:D 12:12, typical colonies were photographed, followed by estimating the diameter of each colony as a growth index with two measurements taken perpendicular to each other across the center.

The spotting method was used to initiate colony growth on CDA plates alone (control) or supplemented with menadione (0.03 mM) or H_2_O_2_ (2 mM) for oxidative stress, Congo red (6 μg/mL) or calcofluor white (5 μg/mL) for cell wall perturbing stress, and NaCl (0.8 M), KCl (0.8 M) or sorbitol (1 M) for osmotic stress, respectively. The diameters of all colonies incubated for 7 day at 25°C were assessed as aforementioned. Cell sensitivity to heat shock was observed with normal 2-day-old SDAY colonies exposed to 42°C for 6 h and 9 h. After exposure, the colonies were transferred to 25°C for a 5-day growth recovery. Typical colonies grown under the stresses were photographed. Relative growth inhibition (RGI) of each strain under each stress was estimated as an index of its sensitivity to each stress cue using the formula RGI = (*d*_c_ – *d*_s_)/*d*_c_ × 100 (*d*_c_, control colony diameter; *d*_s_, stressed colony diameter).

### Assays for conidial yield, submerged blastospore yield, and spore quality.

For assessment of conidiation capacity, 100 μL aliquots of a 10^7^ conidia/mL suspension were evenly spread on SDAY plates (9 cm diameter) and incubated for 12 days at the optimal regime of 25°C and L:D 12:12. For day 3 onwards, a cork borer (5 mm diameter) was used to take three samples daily from each plate culture. Conidial yield in each sample was quantified as the number of conidia per square centimeter of plate culture as described previously (36). Meanwhile, biomass levels were assessed from the cellophane-overlaid SDAY cultures initiated at the same regime. The quality of conidia collected from the cultures of each strain was assayed as the indices of hydrophobicity in an aqueous-organic system, GT_50_ (h) indicative of viability at 25°C, LT_50_ (min) indicative of tolerance to a wet-heat stress at 45°C, and LD_50_ (J/cm^2^) indicative of resistance to UVB irradiation (weighted wavelength: 312 nm), as described elsewhere (56, 70, 79).

To quantify blastospore yield from submerged cultures, 100 mL aliquots of a 10^6^ conidia/mL suspension in TPB, a medium mimicking insect hemolymph and amended from CDB (i.e., agar-free CDA) with 3% trehalose as sole carbon source and 0.3% peptone as sole nitrogen source, were incubated for 5 days on the shaking bed at 25°C. From the end of a 2-day incubation onwards, blastospore concentration and biomass level (mg/mL) were measured daily from the cultures to estimate dimorphic transition rate (no. blastospores/mg biomass).

For each strain, mean size and complexity (density) of conidia used for initiation of TPB culture and of blastospores collected from the 3-day-old TPB cultures were assessed with the FSc and SSc readings from flow cytometry of 2 × 10^4^ conidia or blastospores per sample (three samples per strain). Moreover, conidia and blastospores were labeled with the Alexa Fluor 488-labeled lectins ConA, WGA, PNA, and GNL (Vector Laboratories, Burlingame, CA, USA), followed by FACS analysis to assess the contents of hydrocarbon epitopes on the surfaces of 2 × 10^4^ labeled conidia or blastospores with an argon laser at the excitation/emission wavelengths of 488/530 (±15) nm in the flow cytometer FC 500 MCL (Beckman Coulter, CA, USA). To assess cell wall fragility, 100 mg samples of fresh cells from the TPB cultures were suspended in 2 mL aliquots of 1.0 M NaCl containing snailase and lysing enzymes (Sigma-Aldrich) of 10 mg/mL, followed by shaking incubation for 3, 6, and 9 h of cell wall lysing at 37°C. The concentration of protoplasts released from each of the cell samples was assessed with a hemocytometer.

### Assays for virulence and analysis of virulence-related cellular events.

The virulence of each strain was assayed on G. mellonella larvae (fifth instar) in two infection modes. To initiate NCI, a group of ~35 larvae (three groups per strain) was immersed for 10 s in 40 mL of a 10^7^ conidia/mL suspension. For CBI, 5 μL of a 10^5^ conidia/mL suspension was injected into the hemocoel of each larva in each of three groups. All inoculated groups for NCI or CBI were maintained at 25°C for up to 16 days and monitored for their survival/mortality records at a 12-h (CBI) or 24-h (NCI) interval. The time-mortality trend in each group was subjected to modeling analysis for the estimation of LT_50_ (d) as a virulence index via NCI or CBI.

Several cellular events essential for NCI and hemocoel colonization were examined or analyzed. As a trait critical for initiation of NCI, conidial adherence to insect cuticle was assessed on locust (*Locusta migratoria manilensis*) hind wings pretreated in 37% H_2_O_2_ as described previously (55). Briefly, 5 μL aliquots of a 10^7^ conidia/mL suspension in sterile water free of any surfactant, which may interfere with physical traits of conidial surfaces, were spotted on the central areas of hind wings attached to 0.7% water agar. After an 8-h incubation at 25°C, counts of conidia were made from three microscopic fields of each wing before and after less-adhesive conidia were washed for 30 s in sterile water. Percent ratios of postwash versus prewash counts were computed as an index of conidial adherence to the wing cuticle for each mutant strain with respect to the WT standard. Because cuticular penetration crucial for successful NCI relies upon the actions of extracellular (proteolytic, chitinolytic, and lipolytic) enzymes (ECEs) and Pr1 proteases (49, 50), total ECEs and Pr1 activities (U/mL), and biomass accumulation levels were quantified, respectively, from the supernatants and the 72-h-old cultures generated by shaking 50 mL aliquots of a 10^6^ conidia/mL suspension in CDB containing 0.3% bovine serum albumin (BSA) as sole nitrogen source to induce enzyme production, as described previously (50, 80). In addition, mycosis-killed larvae were maintained at optimal 25°C and monitored for hyphal outgrowths and aerial conidiation on cadaver surfaces in order to reveal an ability for intrahemocoel hyphae of killed larvae to penetrate the host cuticle for outgrowth. The ability reflects a capability of hyphal invasion into insect body via cuticular penetration.

The status of host hemocoel colonization by each strain was examined by observing hemolymph samples of surviving larvae under a microscope to reveal the presence/absence and abundance of hyphal bodies (i.e., blastospores) at the ends of 72-h and 120-h post-CBI. Such hyphal bodies are usually formed by the hyphae arrived in the host hemocoel through dimorphic transition under the control of key CDP activators (38), proliferate rapidly by yeast-like budding until host mummification to death and, hence, reflect the status of fungal hemocoel colonization and killing action (36, 59). Concentration of hyphal bodies in each of three hemolymph samples per larva (three larvae per strain) was assessed daily with a hemocytometer during a period of 48-h to 120-h post-CBI.

### Transcriptional profiling.

For all mutant and WT strains, cellophane-overlaid SDAY and submerged TPB cultures were initiated as aforementioned and incubated for 7 and 5 days at the optimal regime, respectively. From the end of a 48-h incubation onwards, total RNA was extracted daily from each of the SDAY or TPB cultures under the action of RNAiso Plus Kit (TaKaRa, Dalian, China), and reversely transcribed into cDNA under the action of PrimeScript RT reagent kit (TaKaRa). The cDNA samples derived from three independent cultures of each strain on each sampling occasion were used as templates in qPCR analysis with paired primers to assess: (i) transcript levels of *flrA* in the 3-day-old SDAY and TBP cultures of the WT and Δ*flrA* strains; (ii) transcript levels of *flrA* and *fluG* in the 2- to 7-day-old SDAY cultures of the WT strain and in the 3-day-old SDAY and TPB cultures of the WT and DD mutant strains; (iii) daily transcript levels of UDAP (*fluG* and *flbA−E*) and CDP (*brlA*, *abaA*, *wetA* and downstream *vosA*) genes in the SDAY and TPB cultures of all tested strains; and (iv) transcript levels of phenotype-related genes in the 3-day-old SDAY cultures of all tested strains. The phenotype-related genes analyzed were those encoding components of three MAPK (Fus3, Hog1, and Slt2) signaling cascades, five superoxide dismutases (Sod1 to 5), six catalases (Cat1 to 6), and five hydrophobin or hydrophobin-like proteins (Hyd1 to 5). The SYBR Premix *Ex Taq* kit (TaKaRa) was used to perform qPCR analysis with paired primers (Tables S1 and S2). The transcript of the fungal β-actin gene was used as a reference. A threshold-cycle (2^−ΔΔCt^) method was used to compute relative transcript levels for: (i) *flrA* in the 3-day-old SDAY and TBP cultures of the SD mutants relative to the WT strain; (ii) *flrA* and *fluG* in the daily WT cultures with respect to the standard level on day 2 or in the 3-day-old SDAY and TBP cultures of the DD mutants relative to the WT strain; (iii) the UDAP and CDP genes in the daily SDAY and TPB cultures of all mutants relative to the WT strain; and (iv) phenotype-related genes in the 3-day-old SDAY cultures of all mutants with respect to the WT standard. One-fold transcript change was considered as a significant down- or upregulation level of analyzed genes in the mutants versus WT cultures.

### Transcriptomic analysis.

For in-depth insight into similar phenotypes of Δ*flrA* and Δ*fluG*, three 3-day-old cultures (replicates) of the Δ*flrA*, Δ*fluG*, and WT strains grown on cellophane-overlaid SDAY plates at the optimal regime were prepared as aforementioned and sent to Lianchuan BioTech Co. (Hangzhou, China) for construction and analysis of transcriptomes as described previously (56). Clean tags gained by filtration of all raw reads from sequencing on an Illumina Novaseq 6000 platform were normalized as fragments per kilobase of exon per million fragments mapped (FPKM) and mapped to the B. bassiana genome (51). DEGs were identified at the significant levels of both log_2_ *R* (fold change) ≤ −1 (downregulated) or ≥1 (upregulated) and *q *< 0.05 and annotated with known or putative gene information in the NCBI protein databases, followed by GO analysis (http://www.geneontology.org/) for enrichments of GO terms to three function classes (*P < *0.05) and KEGG analysis (http://www.genome.jp/kegg/) for pathway enrichment (*P < *0.05).

The Δ*flrA*- and Δ*fluG*-specific transcriptomes were validated by comparing relative transcript levels of 16 selected genes in the cDNA samples derived from the 3-day-old SDAY cultures of Δ*flrA* and Δ*fluG* versus WT with their anti-log_2_ *R* values in the two transcriptomes, respectively. The transcript levels of two CDP genes (*brlA* and *abaA*), two UDAP genes (*flbB* and *flbD*), six kinase genes (Hog1 and Slt2 cascades), and six catalase genes (*cat1* to *6*) were quantified via the qPCR analysis as aforementioned or described previously (36).

### Statistical analysis.

All experimental data were subjected to one-way analysis of variance and Tukey’s honestly significant difference (HSD) test for phenotypic differences among the WT and SD/DD mutant strains.

### Data availability.

All data generated or analyzed during this study are included in the paper and associated supplemental files. All RNA-seq data analyzed in this study are available at the NCBI’s Gene Expression Omnibus under the accession GSE193058 (https://www.ncbi.nlm.nih.gov/geo/query/acc.cgi?acc=GSE193058) aside from those reported in Tables S4 to S7 of this paper.
