# Supplementary figures and images for: FluG and FluG-like FlrA Coregulate Manifold Gene Sets Vital for Fungal Insect-Pathogenic Lifestyle but Not Involved in Asexual Development
Source: mSystems. 2022 Jul 11;7(4):e00318-22. doi: 10.1128/msystems.00318-22 (PMC9426541; doi:10.1128/msystems.00318-22)

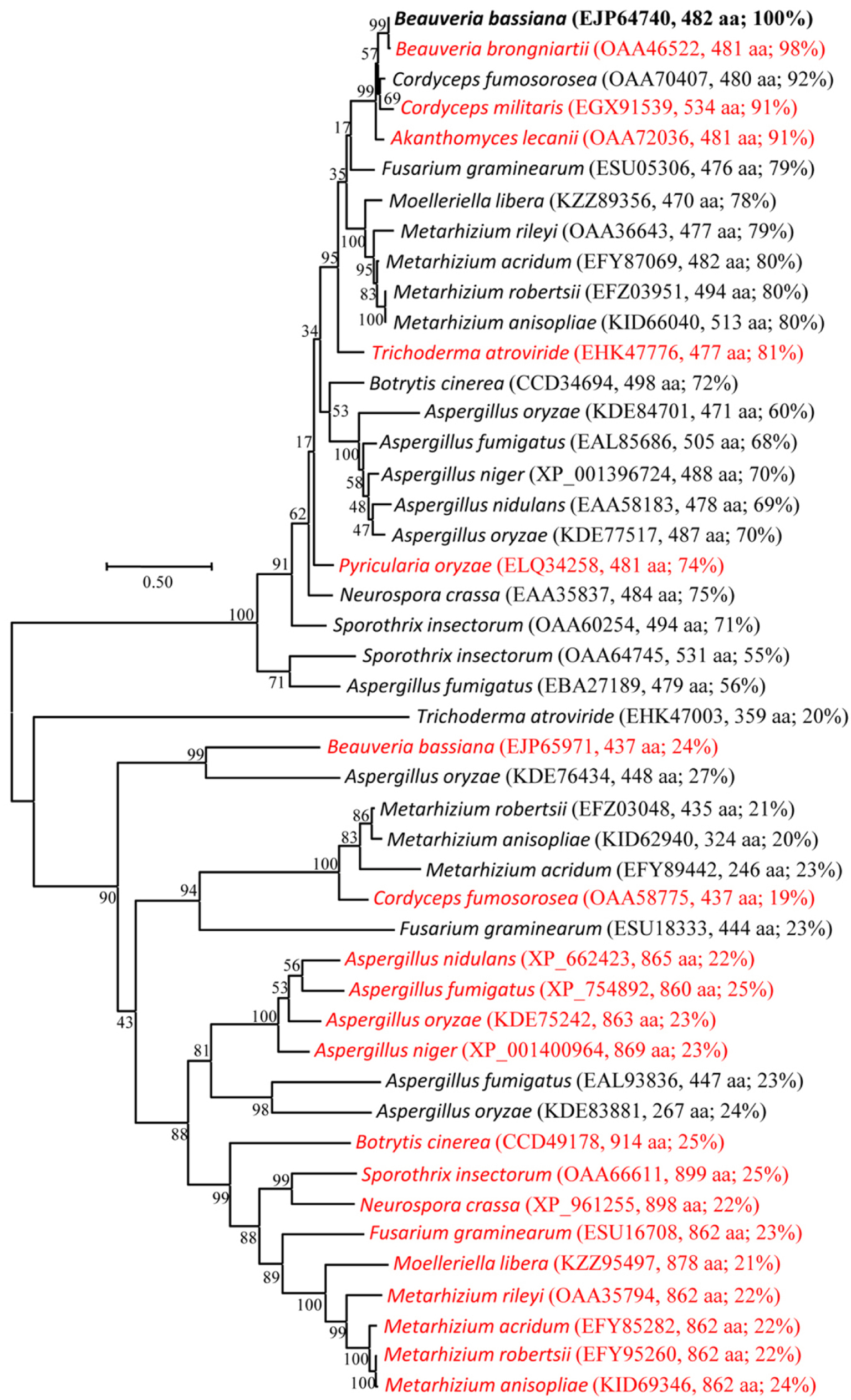

Supplement: FIG S1 [file msystems.00318-22-s0001.jpg]

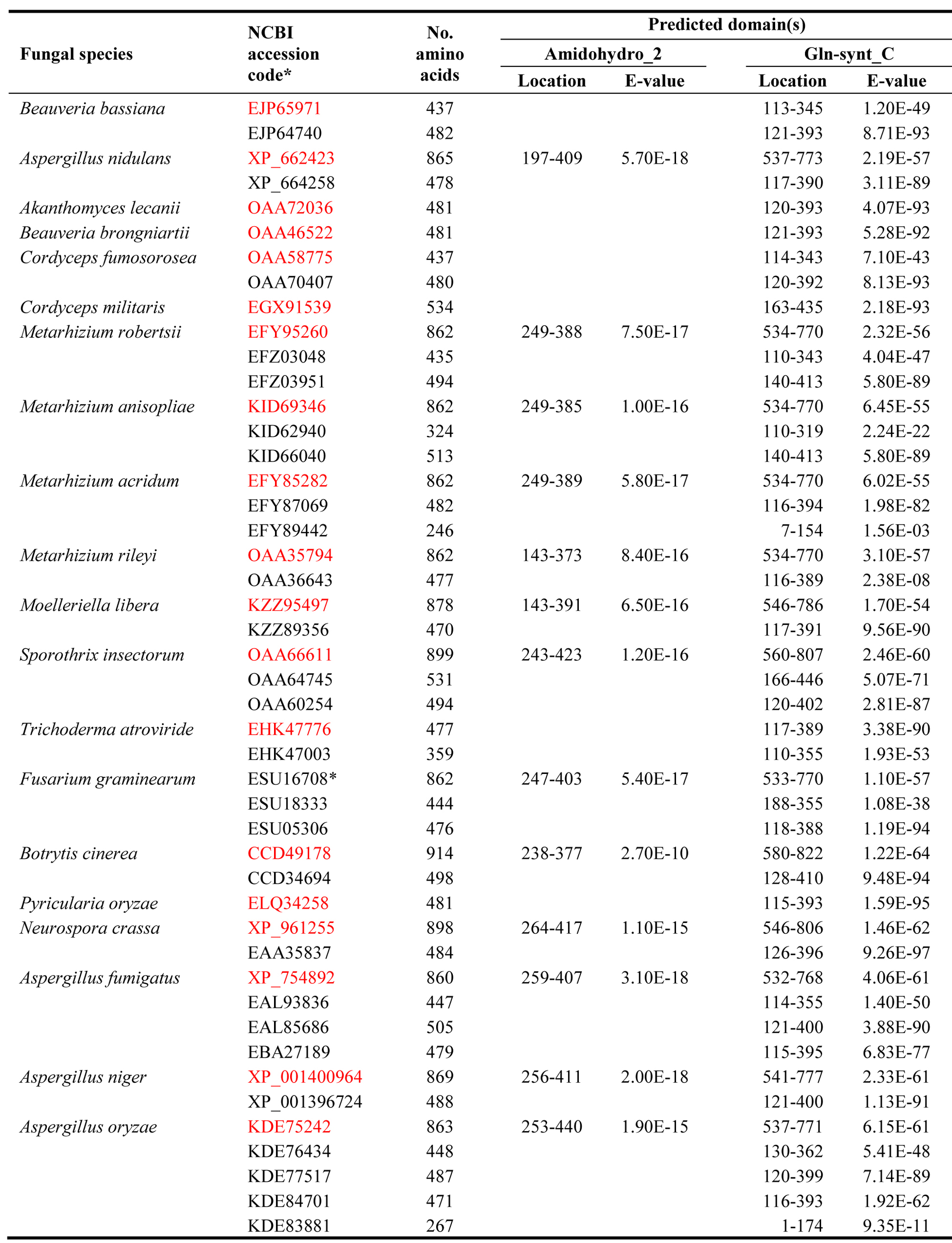

Supplement: TABLE S1 [file msystems.00318-22-s0004.jpg]

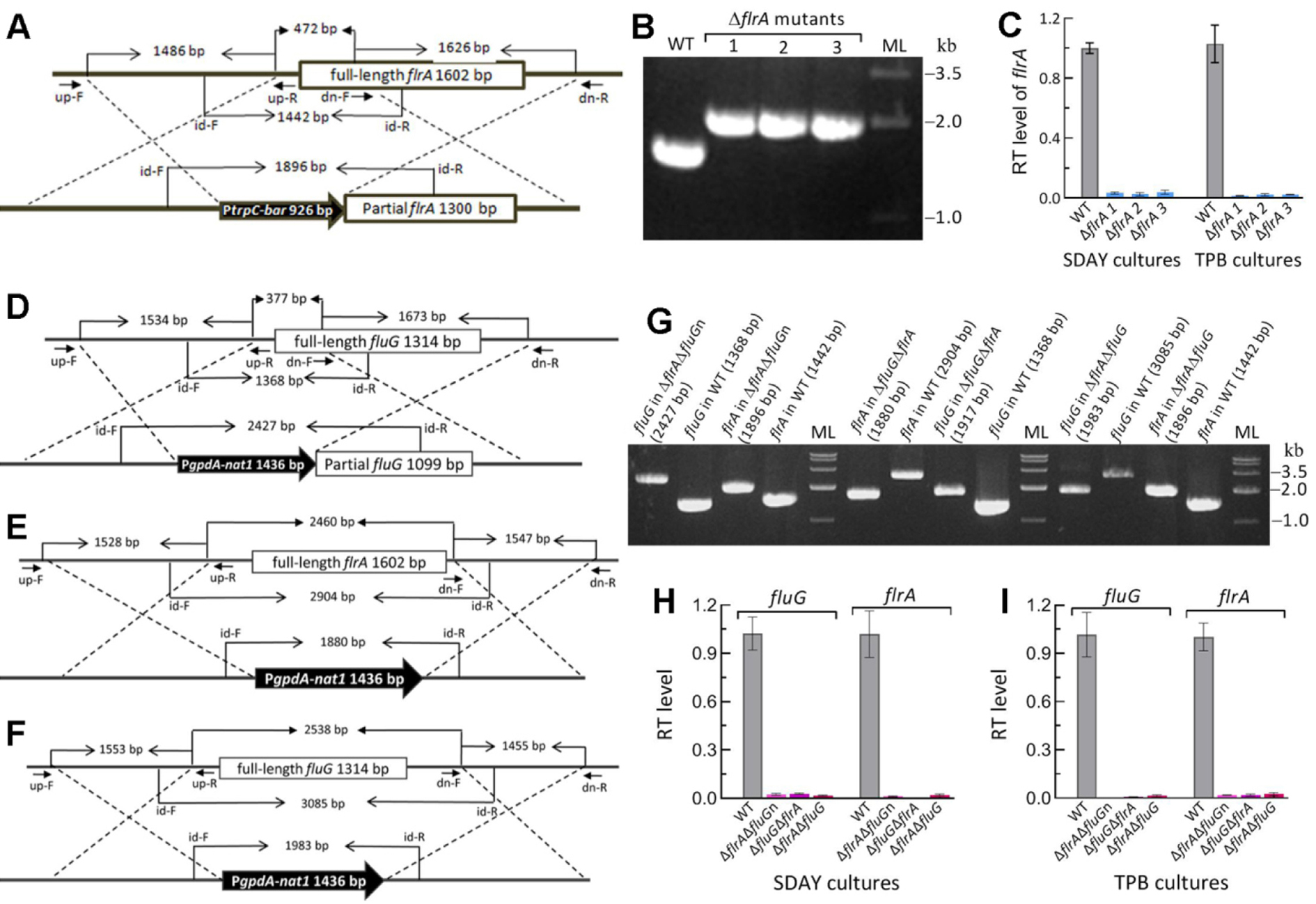

Supplement: FIG S2 [file msystems.00318-22-s0002.jpg]

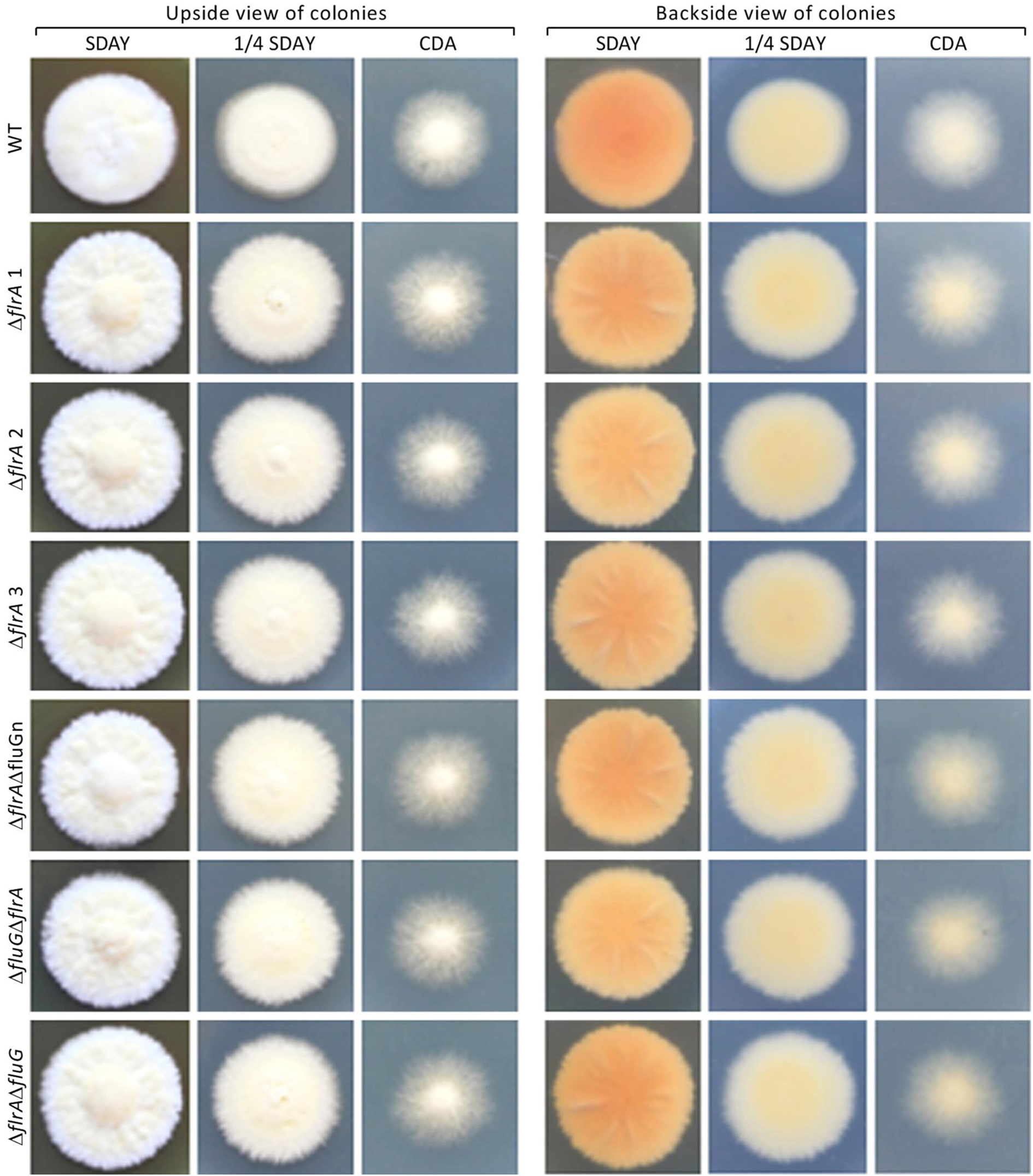

Supplement: FIG S3 [file msystems.00318-22-s0003.jpg]

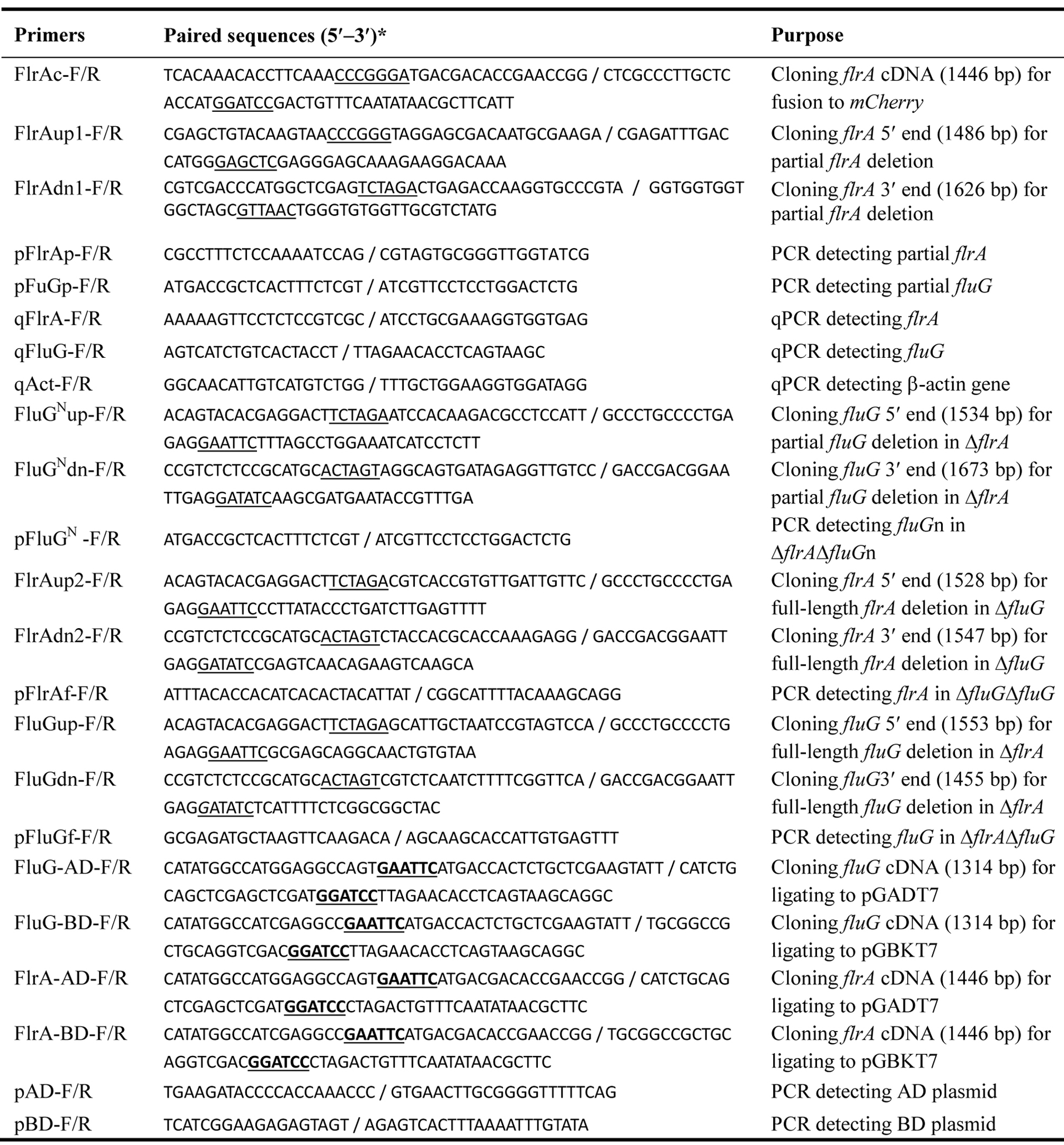

Supplement: TABLE S2 [file msystems.00318-22-s0005.jpg]

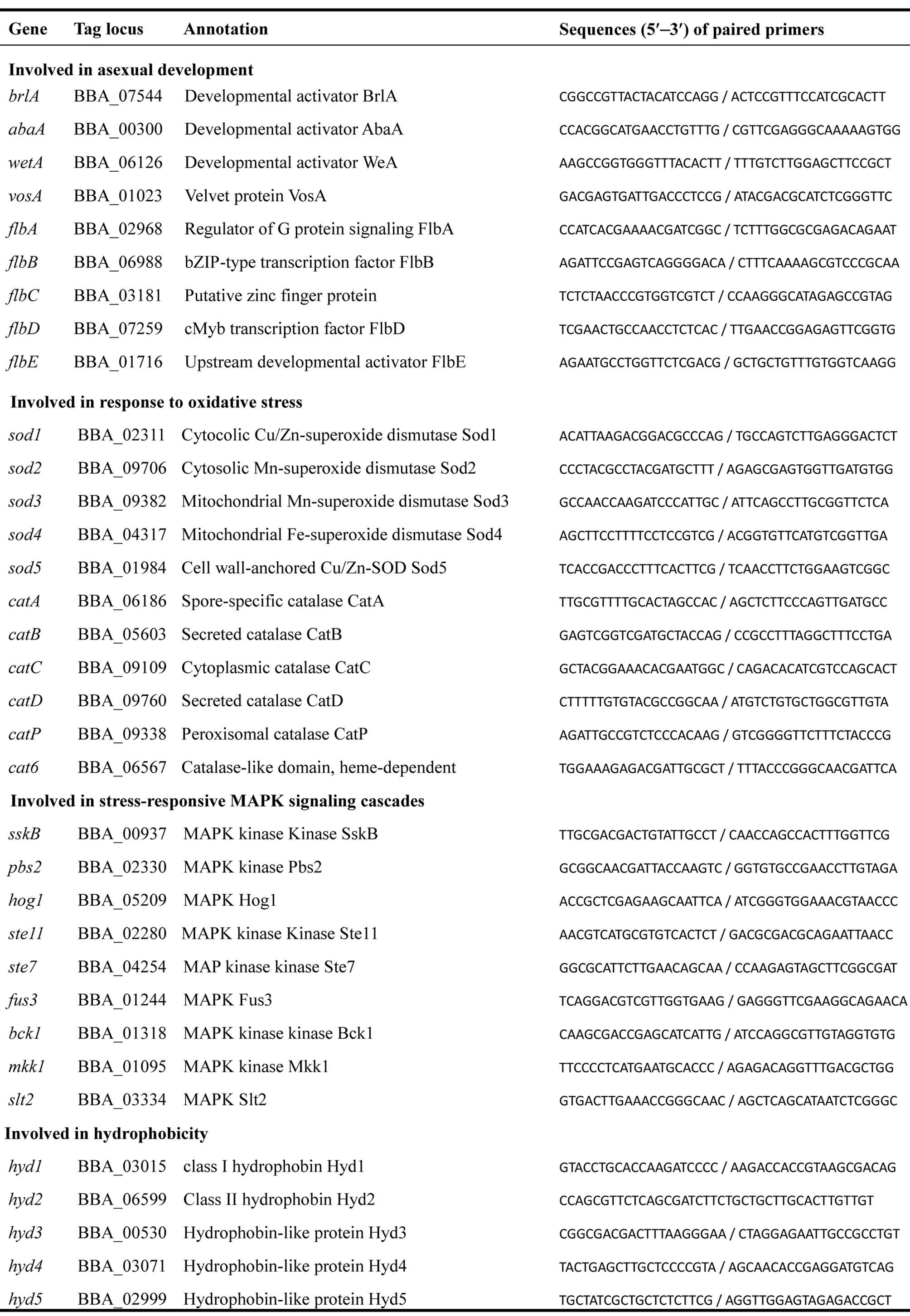

Supplement: TABLE S3 [file msystems.00318-22-s0006.jpg]
